# Phase separation as a possible mechanism for dosage sensitivity

**DOI:** 10.1186/s13059-023-03128-z

**Published:** 2024-01-15

**Authors:** Liang Yang, Jiali Lyu, Xi Li, Gaigai Guo, Xueya Zhou, Taoyu Chen, Yi Lin, Tingting Li

**Affiliations:** 1 Department of Medical Bioinformatics, School of Basic Medical Sciences, Peking University Health Science Center, Beijing, 100191 China; 2 IDG/McGovern Institute for Brain Research, Tsinghua-Peking Joint Centre for Life Sciences, School of Life Sciences, Tsinghua University, Beijing, 100084 China; 3 Department of Systems Biology, Columbia University, New York, NY 10032 USA; 4 Key Laboratory for Neuroscience, Ministry of Education/National Health Commission of China, Peking University, Beijing, 100191 China

## Abstract

**Background:**

Deletion of haploinsufficient genes or duplication of triplosensitive ones results in phenotypic effects in a concentration-dependent manner, and the mechanisms underlying these dosage-sensitive effects remain elusive. Phase separation drives functional compartmentalization of biomolecules in a concentration-dependent manner as well, which suggests a potential link between these two processes, and warrants further systematic investigation.

**Results:**

Here we provide bioinformatic and experimental evidence to show a close link between phase separation and dosage sensitivity. We first demonstrate that haploinsufficient or triplosensitive gene products exhibit a higher tendency to undergo phase separation. Assessing the well-established dosage-sensitive genes HNRNPK, PAX6, and PQBP1 with experiments, we show that these proteins undergo phase separation. Critically, pathogenic variations in dosage-sensitive genes disturb the phase separation process either through reduced protein levels, or loss of phase-separation-prone regions. Analysis of multi-omics data further demonstrates that loss-of-function genetic perturbations on phase-separating genes cause similar dysfunction phenotypes as dosage-sensitive gene perturbations. In addition, dosage-sensitive scores derived from population genetics data predict phase-separating proteins with much better performance than available sequence-based predictors, further illustrating close ties between these two parameters.

**Conclusions:**

Together, our study shows that phase separation is functionally linked to dosage sensitivity and provides novel insights for phase-separating protein prediction from the perspective of population genetics data.

**Supplementary Information:**

The online version contains supplementary material available at 10.1186/s13059-023-03128-z.

## Background

In the human genome, gene deletion (haploinsufficiency) or duplication (triplosensitivity) results in changes in gene dosage, while dosage changes in dosage-sensitive genes would result in phenotypic effects [1–3]. More than 300 haploinsufficient genes as well as 13 triplosensitive genes have been reported in human, which are associated with a variety of disorders such as neurodevelopmental disorders [4]. Recently, using machine-learning, a gene map was generated for dosage-sensitive genes from copy number variant data of nearly one million individuals. This map contains nearly 3000 haploinsufficient and over 1500 triplosensitive genes [3]. However, it is unclear why a subset of genes exhibit abnormal phenotypes upon gene dosage changes while the majority of others do not. The general mechanisms underlying dosage sensitivity remain poorly understood.

Phase separation is a natural process occurring intracellularly that compartmentalizes protein, RNA as well as DNA in a concentration-dependent manner. During the phase separation process, these biomolecules assemble into separated condensates provided that these molecules are present at concentrations above a critical threshold [5, 6]. High concentrations of specific components within these phase-separated condensates allow biological reactions to occur at accelerated rates, as these condensates enrich relevant molecules and exclude non-relevant or inhibitory molecules [5]. Therefore, dosage-sensitive gene products and phase-separating proteins share similar concentration-dependent properties.

In addition to concentration dependency, products of dosage-sensitive genes and phase-separating proteins share other characteristics. For instance, intrinsically disorder regions (IDRs) and interaction domains/motifs within protein sequences assist in predicting dosage-sensitive genes [7], while interactions mediated by IDRs and multiple interaction domains/motifs constitute the driving forces of the processes behind phase separation. Furthermore, the protein products of dosage-sensitive genes tend to form homodimers [1], and phase-separating proteins often possess dimerization or oligomerization domains which are essential for the multivalent interactions to drive the phase separation process [8]. Dosage-sensitive gene products and phase-separating proteins are enriched in similar biological pathways such as transcription regulation, RNA splicing, and signaling pathway [6, 9]. Lastly, dosage-sensitive genes lose their functions when under- or over-expressed [10]; similarly, phase-separating proteins result in abnormal protein assembly and cellular toxicity when abnormally expressed [11, 12]. These similarities suggest that dosage sensitivity and phase separation are functionally related. However, little evidence supports this hypothesis to date.

Several dosage-sensitive gene products have previously been reported to undergo phase separation, including MECP2, SYNGAP1, SOX2, and PAK2 [6, 13–16]. However, so far only one recent study directly investigated the link between phase separation and dosage sensitivity [17]. In that study, loss-of-function (LoF) mutation in KMT2D was shown to impair its normal phase separation process owing to decreased KMT2D protein concentration, altering the functional partitioning of chromatin. As a result, patients carrying this mutation suffer from the haploinsufficiency-related disease named Kabuki syndrome [17]. Systematic studies to investigate the relationship between dosage sensitivity and phase separation are urgently required.

In this study, both computational analysis and biological experiments showed that dosage-sensitive gene products exhibit a higher tendency to undergo phase separation. We then experimentally introduced pathogenic variations to dosage-sensitive genes to investigate whether dosage insufficiency leads to defect in phase separation. Furthermore, we utilized multi-omics data analysis to explore whether LoF genetic perturbations on phase-separating genes cause disturbed phenotypes. In addition, most of the current phase separation predictors rely on sequence features, and the prediction performance needs further improvement [18]. Based on the close ties between dosage sensitivity and phase separation, we developed an efficient phase separation predictor based on dosage-sensitive scores derived from population genetics data.

## Results

### Dosage-sensitive gene products tend to undergo phase separation

**Fig. 1:**
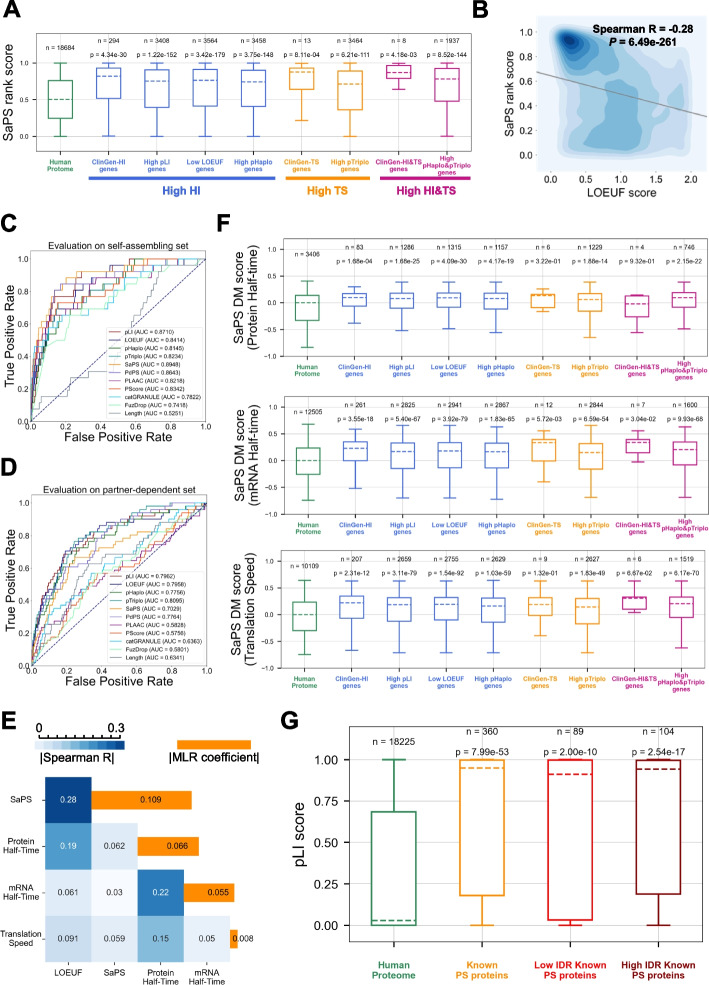
Dosage-sensitive gene products tend to undergo phase separation. **A** Comparison of SaPS rank score between human proteome and gene sets with high dosage sensitivity. HI/TS genes: haploinsufficient/triplosensitive gene products from ClinGen. High pLI/pHaplo/pTriplo genes: genes with top 20% pLI/pHaplo/pTriplo scores. Low LOEUF genes: genes with bottom 20% LOEUF scores. *P*-value was calculated with the two-sided Mann–Whitney *U* test. **B** Kernel density regression plot of LOEUF score and SaPS rank score. The coefficient is represented with the Spearman correlation coefficient. *P*-value was calculated with Spearman’s rank correlation test. **C**, **D** AUC performance of predicting self-assembling (**C**) and partner-dependent phase-separating proteins (**D**) on the test set. **E** Comparison of SaPS DM score between the human proteome and gene sets with high dosage sensitivity. *P*-value was calculated with the two-sided Mann–Whitney *U* test. **F** Heatmap of Spearman correlation coefficient between LOEUF score and factors. The bar represents the multiple regression coefficients between LOEUF score and factors. **G** Comparison of pLI score in proteins sets. Disordered regions were calculated with the ESpritz DisProt program. *P*-value was calculated with the two-sided Mann–Whitney *U* test. PS: phase-separating

To better understand whether dosage-sensitive gene products exhibit a general tendency to undergo phase separation, we assessed the phase separation scores of dosage-sensitive genes using our previously developed phase separation predictor SaPS [19]. As a result, we obtained 311 haploinsufficient and 13 triplosensitive genes from the ClinGen database [20]. Compared to proteins in the human proteome, the scores for phase separation were significantly higher for dosage-sensitive gene products (Fig. 1A). Furthermore, we found that eight dosage-sensitive genes belonging to both haploinsufficient and triplosensitive genes exhibited the highest phase separation scores (Fig. 1A). Since the number of verified dosage-sensitive genes remains limited, we extended our analysis to genes predicted to have high dosage sensitivity potential. Haploinsufficient genes tend to be LoF-intolerant, and two LoF-intolerance scores, namely pLI [21] and LOEUF [22], were used to identify genes with high haploinsufficiency potential. In addition, the pHaplo and pTriplo score generated from large-scale copy number variant data were used to identify genes with high haploinsufficient/triplosensitive potential [3]. As shown in Fig. 1A, genes exhibiting high dosage sensitivity potentials (corresponding to high pLI scores, low LOEUF scores, high pHaplo scores, and high pTriplo scores) exhibited significantly higher phase separation scores as well.

Next, we examined whether dosage-sensitive scores are significantly correlated with phase separation scores using linear regression. As shown in Fig. 1B and Additional file 1: Fig. S2, both haploinsufficient and triplosensitive measures were significantly correlated with phase separation scores. Currently, multiple phase separation predictors are available, with each algorithm preferentially prioritizing phase-separating proteins featuring different sequence information [18]. We repeated the correlation analysis using phase separation scores generated by other available predictors, including PLAAC [23], PScore [24], catGRANULE [11], and FuzDrop [25]. As shown in Additional file 1: Fig. S1-S2, phase separation scores generated by all these predictors were significantly correlated with dosage-sensitive scores as well. As available phase separation predictors do not rely on population genetics information used in dosage-sensitive scores, we were confident that the close relationship between dosage sensitivity and phase separation was not caused by the confounding bias from population genetics information or protein sequence information.

Based on these findings, we hypothesized that dosage-sensitive scores can be used to measure the ability of proteins to phase separate. To validate our hypothesis, we built the phase separation predictors based on dosage-sensitive scores and evaluated the predictors by area under the curve (AUC). Phase-separating proteins can be divided into two groups: self-assembling proteins, which can phase separate spontaneously, and partner-dependent proteins, which interact with partners to undergo phase separation [19]. For self-assembling proteins (Additional file 2: Table S1), the phase separation predictors based on dosage-sensitive scores achieved well AUC performance (Fig. 1C). For partner-dependent proteins (Additional file 2: Table S1), except PdPS which integrated posttranslational modification information, most of the available phase separation predictors performed poorly (Fig. 1D). However, the phase separation predictors based on dosage-sensitive scores exhibited much higher AUC than most of available phase separation predictors (Fig. 1D).

Previous researches have demonstrated that dosage sensitivity is correlated with a number of factors such as protein half-life, mRNA half-life, and translation rate (Additional file 1: Fig. S3A-C) [26–29]. To control the impact of other factors, we applied distance to the median (DM) score to standardize each factor respectively. The results showed that phase separation score is still significantly higher for genes with high dosage sensitivity (Fig. 1E). We also performed multiple linear regression analysis in order to control multiple factors simultaneously, which also showed that the coefficient of phase separation to dosage sensitivity is the highest when we kept other factors consistent (Fig. 1F). These in silico analysis demonstrated that dosage sensitivity highly correlated with phase separation independent of other related factors.

Researches have also shown that promiscuous linear motifs in disordered regions are associated with dosage sensitivity [7]. Moreover, disordered regions in proteins are important mediators of phase separation. In order to exclude the possibility that promiscuous linear motifs in the disordered regions might be the cause of the association between phase separation and dosage sensitivity, we compared the dosage sensitivity score between the known phase-separating proteins with high and low proportions of disordered regions. Compared to proteins in the human proteome, the scores for dosage sensitivity were significantly higher both for phase-separating proteins with high and low proportions of disordered regions (Fig. 1G). Therefore, this result demonstrated that dosage sensitivity is highly correlated with phase separation independent of disordered region proportions.

### Protein products of the dosage-sensitive genes PQBP1, HNRNPK, and PAX6 undergo phase separation

**Fig. 2:**
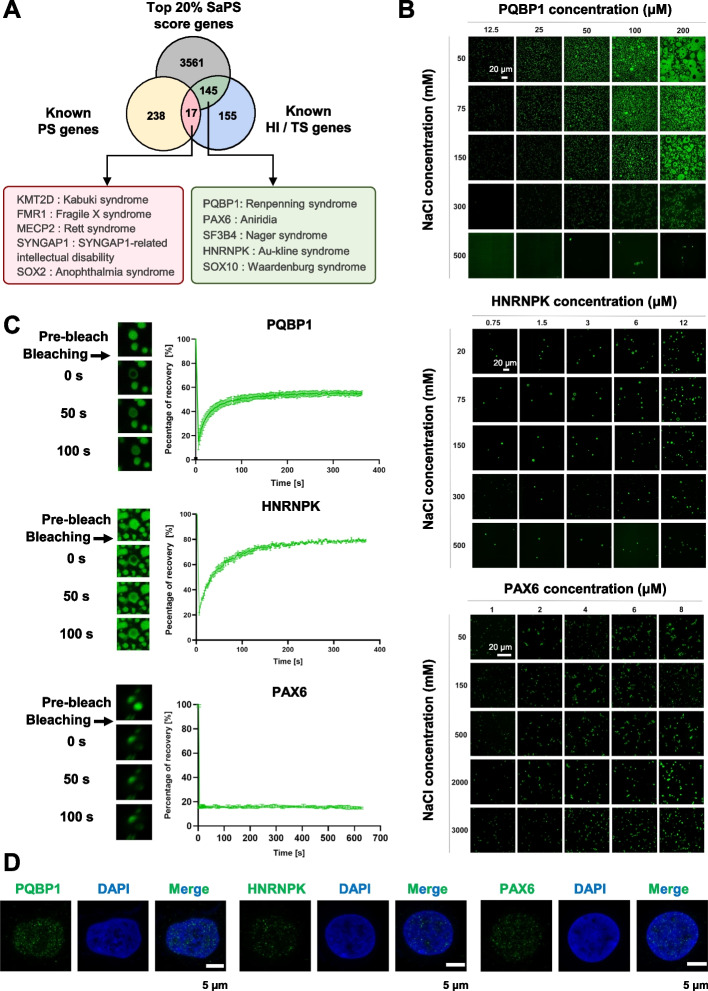
Dosage-sensitive genes PQBP1, HNRNPK and PAX6 products undergo phase separation. **A** Venn diagram among known phase-separating (PS) proteins from PhaSepDB, proteins with top 20% SaPS in the human proteome and known haploinsufficient (HI)/triplosensitivity (TS) gene products from ClinGen. **B** Phase diagrams of PQBP1, HNRNPK and PAX6 with different NaCl concentrations. Scale bars, 20 µm. **C** FRAP of the droplets formed in vitro by PQBP1 and HNRNPK in the presence of 150 mM NaCl, and PAX6 in the presence of 2 M NaCl. *n* = 3 biologically independent samples, data are presented as mean values ± SEM. **D** Confocal images of endogenous PQBP1 (HEK 293T cells), HNRNPK (HeLa cells), and PAX6 (HeLa cells) in wild-type cells. Cells were stained with antibodies of target proteins (green) and DAPI (blue). Scale bars, 5 µm

Of the 317 dosage-sensitive genes from the ClinGen database, 17 gene products were previously reported to undergo phase separation, such as KMT2D [17], SYNGAP1 [14], and SOX2 [15] (Fig. 2A, Additional file 1: Fig. S4). Many dosage-sensitive gene products exhibit high phase separation scores but have not been verified experimentally (Fig. 2A). Thus, we tested the phase separation ability of the three proteins, PQBP1 in Renpenning syndrome [30], HNRNPK in Au-Kline syndrome [31], and PAX6 in Aniridia [32]. We purified the bacterially expressed recombinant PQBP1, HNRNPK, and PAX6 proteins (Additional file 1: Fig. S5) and analyzed their phase separation in vitro. As shown in Fig. 2B, both PQBP1 and HNRNPK formed spherical liquid droplets, in a salt and protein concentration-dependent manner (Fig. 2B). Next, we used fluorescence recovery after photobleaching (FRAP) to quantify the droplet’s fluidity. PQBP1 droplets showed a recovery of 50% fluorescence intensity within 90 s post-bleaching. Similarly, HNRNPK droplets reached 50% recovery within 30 s, indicating a highly dynamic exchange of both proteins between the droplets and the environment (Fig. 2C).

PAX6 condensed under conditions at low protein concentration and extremely high salt concentration, together with exhibiting a very slow recovery rate (Fig. 2B, C). A similar case was reported before. SOX2, another pluripotent transcription factor, forms droplets only at low protein concentration and high salt concentration [15]. PAX6 and SOX2 form SOX2/PAX6/DNA ternary complex and together promote lens development [33]. Haploinsufficiency of either PAX6 or SOX2 results in similar eye diseases [32, 34]. Together, these similar phase separation behaviors suggested that PAX6 is functionally related to SOX2.

We also purified truncated proteins as control groups, including recombinant HNRNPK-IDR (residues 269–463), HNRNPK-∆IDR (residues 1–268), PAX6-IDR (residues 173–422), PAX6-∆IDR (residues 1–172), PQBP1-IDR (residues 142–265), and PQBP1-∆IDR (residues 1–141) proteins to test their phase separation ability. The IDR regions of these proteins undergo phase separation significantly, forming spherical droplets, but their ∆IDR regions did not form spherical droplets. HNRNPK-∆IDR and PQBP1-∆IDR formed precipitates and PAX6-IDR was in a soluble state (Additional file 1: Fig. S5-6). These truncated proteins prove that the phase separation phenomenon depends on the predicted IDR.

In addition, immunofluorescence experiments for PQBP1, HNRNPK, and PAX6 showed that these proteins formed clear puncta in cells (Fig. 2D). Together, our computational and experimental results suggested that protein products of dosage-sensitive genes undergoing phase separation are more general than currently appreciated.

### Putative LoF mutations in dosage-sensitive genes result in phase separation defect

After showing that dosage-sensitive genes tend to undergo phase separation, we next explored whether pathogenic variations in dosage-sensitive genes would impact their phase separation process. Since phase separation requires protein concentrations to reach a critical level, we hypothesized that products of dosage-sensitive genes would not undergo phase separation if LoF mutations cause protein levels to be below the concentration that triggers phase separation. To test our hypothesis, we searched the ClinVar database [35] for LoF mutations resulting in a decrease in protein level of haploinsufficient genes. Deletion of one copy of haploinsufficient gene results in reduced protein levels, while protein-truncating variants (PTVs) which introduce premature stop codons to haploinsufficient genes might promote degradation of mutant mRNAs by nonsense-mediated mRNA decay (NMD), lowering protein levels eventually as well [36]. Therefore, we defined here deletions and NMD-causing mutations as putative LoF mutations.

**Fig. 3:**
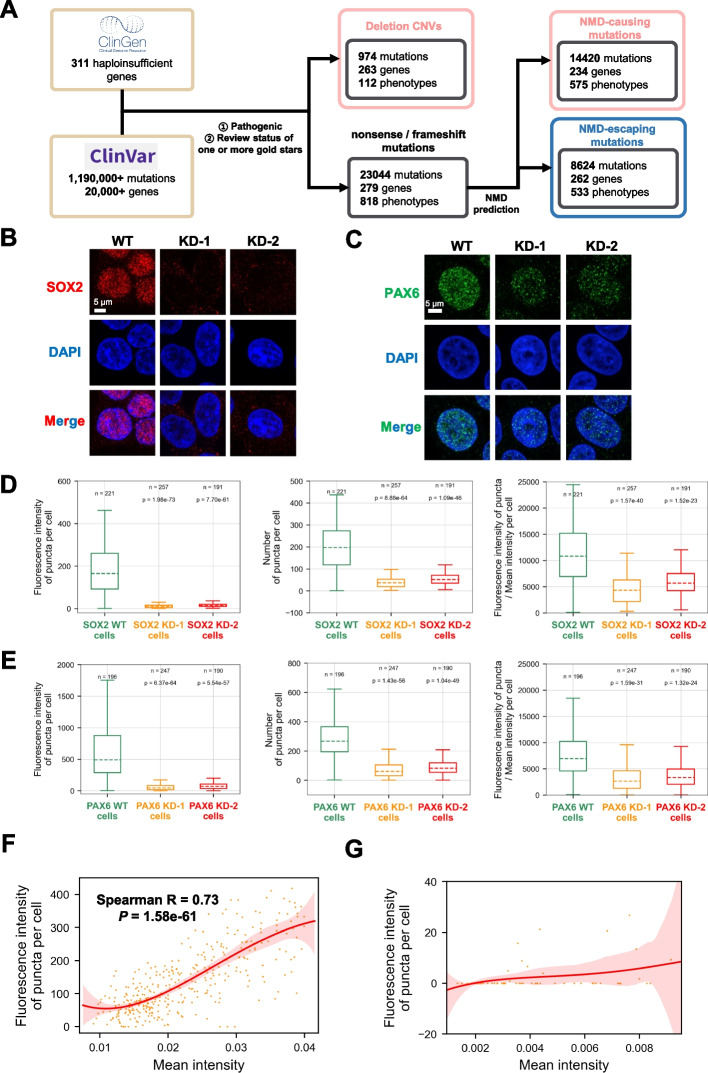
LoF mutations in dosage-sensitive genes destabilize condensate activity in cell. **A** Screening process for disease-associated mutations in haploinsufficient genes in ClinVar database. **B**, **C** Confocal images of endogenous SOX2 or PAX6 in wild-type (WT) cells and knockdown (KD) cells with heterozygous deletion of SOX2 (HEK 293T cells) or PAX6 (HeLa cells). Cells were stained with SOX2 antibody (red), PAX6 antibody (green) and DAPI (blue). Scale bar, 5 µm. **D**, **E** Comparison of fluorescence intensity puncta per cell, number of puncta per cell and fluorescence intensity of puncta/mean fluorescence intensity per cell between wild-type (WT) cells and knockdown (KD) cells. *P*-value was calculated with the two-sided Mann–Whitney *U* test. **F**, **G** Curve of the mean fluorescence intensity of mCherry-PQBP1 (**F**) (*n* = 509)/mCherry-PQBP1-∆IDR (**G**) (*n* = 69) versus fluorescence intensity of puncta per cell. All images are quantified with the same optical settings. The coefficient is represented with the Spearman correlation coefficient. *P*-value was calculated with Spearman’s rank correlation test

Of the 311 haploinsufficient genes defined in ClinGen database, we found 263 genes that harbor pathogenic deletions and 234 genes possessing pathogenic NMD-causing mutations based on rules of NMD-escaping [37] (Fig. 3A, Additional file 1: Fig. S7, Additional file 3: Table S2). For example, it was reported that one gene copy deletion of the haploinsufficient gene SOX2 causes Anophthalmia syndrome, which is characterized by abnormal development of the eyes and other parts of the body [34]. To assess the consequences of gene copy deletion on phase separation, we constructed two heterozygous knockdown cell lines via CRISPR-Cas9-mediated gene editing by targeting one of the two alleles. We found that compared to wild-type cell line, the expression levels of SOX2 or PAX6 in the knockdown lines were lower (Additional file 1: Fig. S8A-B). As shown in Fig. 3B–E and Additional file 1: Fig. S8G-J, SOX2 or PAX6 formed a larger number of puncta in the nucleus of wild-type cells following immunofluorescence staining, while both of two independent knockdown cell lines per gene showed far less puncta. To explore whether phase separation intensity decreases more dramatically than protein concentration in the heterozygous knockdown cells, we normalized the phase separation intensity by mean fluorescence intensity indicating protein concentration in the cells. The results showed that the normalized phase separation intensity in the knockdown cell lines is still lower than that of wild-type cells (Fig. 3D, E). In agreement with our hypothesis, one gene copy deletion of SOX2 or PAX6, which mimicked the heterozygous mutation in diseases, changed their phase separation property *in cell.*

Next, we directly assessed how protein expression of haploinsufficient genes correlated with their phase separation ability. To this end, we transfected PQBP1 knockout cells with plasmids expressing mCherry-PQBP1 and mCherry-PQBP1-∆IDR fusion protein at different doses. We found that the intensity of mCherry-PQBP1 puncta fluorescence displays a non-linear relationship with the concentration of transfected protein (Fig. 3F). As a negative control, mCherry-PQBP1-∆IDR did not form any puncta in cells, which was consistent with the droplet assay experiment (Fig. 3G, Additional file 1: Fig. S5-6). We found no correlation between the formation of droplets and mCherry-PQBP1-∆IDR levels in the negative control. This finding suggested the extent of phase separation primarily relies on protein expression levels of haploinsufficient genes.

### NMD-escaping mutations in dosage-sensitive genes lose phase-separation-prone regions and cause abnormal phase separation

**Fig. 4:**
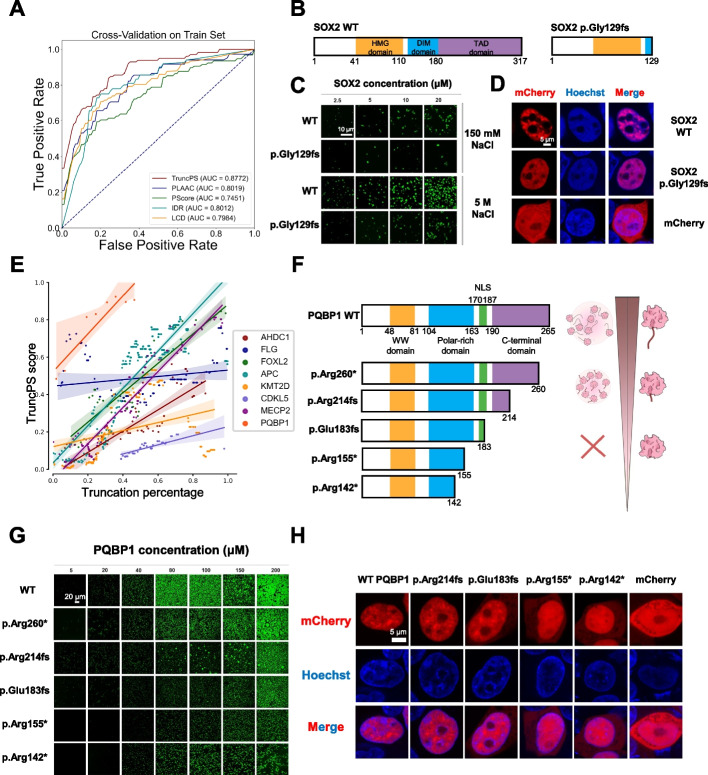
NMD-escaping mutations in dosage-sensitive genes cause abnormal phase separation. **A** AUC performance of predicting phase-separation-prone regions. **B** Schematic diagram of the wild-type and mutant SOX2 protein domains. **C** Phase diagram of wild-type SOX2 protein and NMD-escaping mutant SOX2 protein with 150 mM and 5 M NaCl concentrations. Scale bar, 10 µm. **D** Confocal images of live SOX2 knockdown HEK 293T cells transfected with mCherry tagged wild-type SOX2 protein, NMD-escaping mutant SOX2 protein and mCherry, and stained with Hoechst (blue). Scale bar, 5 µm. **E** Regression plot of mutation truncation percentage and TruncPS score of NMD-escaping mutations in haploinsufficient genes. **F** Left: schematic diagram of the wild-type and mutant PQBP1 protein domains. Right: model of the relationship between protein truncation length and phase separation ability. The longer the truncation length, the weaker the phase separation ability. **G** Phase diagram of PQBP1 NMD-escaping mutant proteins with 150 mM NaCl concentration. Scale bar, 20 µm. **H** Confocal images of live PQBP1 knockout HEK 293T cells transfected with mCherry tagged wild-type PQBP1 proteins, NMD-escaping mutant PQBP1 proteins and mCherry, and stained with Hoechst (blue). Scale bar, 5 µm

In addition to NMD-causing mutations, NMD-escaping mutations failing to trigger NMD commonly result in the expression of truncated proteins [38]. To assess whether the truncated proteins with loss of phase-separation-prone regions exhibit abnormal phase separation ability, we obtained NMD-escaping mutations from the ClinVar database. Of the 311 haploinsufficient genes in ClinGen database, 262 genes harbored pathogenic NMD-escaping mutations (Fig. 3A, Additional file 1: Fig. S7, S9A, Additional file 3: Table S2). To identify truncated proteins which might lose phase-separation-prone regions, we developed a tool called TruncPS to evaluate the phase separation potential of the truncated regions (Additional file 1: Fig. S9B, see Methods). Briefly, TruncPS used experimentally verified phase-separation-prone regions in PhaSepDB [39] as positive training set (Additional file 5: Table S4), and evaluated the phase separation capability of the truncated region, namely by integrating multiple features including sequence embedding, intrinsically disordered region (IDR) proportion, low-complexity domain (LCD) proportion, hydropathy, kappa, and net-charge properties. As shown in Fig. 4A, the prediction performance of TruncPS was much better than currently available phase separation predictors used to screen phase-separation-prone regions.

To obtain phase separation impact score for NMD-escaping mutations, we applied TruncPS on the truncated regions of NMD-escaping mutations in the 262 haploinsufficient genes (Additional file 1: Fig. S9C-D). For example, a frame-shift mutation at position 129 of SOX2 protein results in a loss of 188 aa (Fig. 4B, Additional file 4: Table S3), thus generating a truncated SOX2 version. A high TruncPS score of this mutation indicates that the ability of SOX2 to phase separate reduces following truncation. To validate this prediction, we assessed how the mutation changes phase separation ability of SOX2. As shown in Fig. 4C, the protein-truncating variant SOX2 p.Gly129fs resulted in a significant reduction in phase separation compared to wild-type SOX2 protein, under physiological salt concentration. The contrast was even more significant in the presence of 5 M NaCl (Fig. 4C and Additional file 1: Fig. S5). We complemented SOX2 knockdown cells with similar level wild-type SOX2 or SOX2 p.Gly129fs proteins (Additional file 1: Fig. S11C-E). And we observed the same intracellular results with extracellular results with the same mean fluorescence intensity of proteins (Fig. 4D and Additional file 1: Fig. S10A-B). Together, these findings strongly suggested that the loss of partial phase-separation-prone regions due to the mutation abolishes the ability of SOX2 to phase separate.

Furthermore, a considerable number of haploinsufficient genes possess multiple NMD-escaping mutations which generate truncations with different lengths. As shown in Fig. 4E, generally longer truncated region is coupled with higher TruncPS score. We observed that the length of the truncated region positively correlated with the decrease in phase separation ability. To validate this observation, we generated truncations with different lengths for PQBP1 (Additional file 4: Table S3). PQBP1 consists mainly of a folded WW domain (residues 48–81), the central IDR (residues 104–163) with a polar-amino-acid-rich domain (PRD) of high charge density, followed by a nuclear localization signal (NLS; residues 170–187) and a C-terminal IDR (residues 190–265) (Fig. 4F). The in vitro phase separation ability of a mutant form of PQBP1, namely p.Arg260*, was slightly lower than that of wild-type, while phase separation ability of p.Arg214fs, p.Glu183fs progressively decreased as truncated region lengthened. The p.Arg155* and p.Arg142* mutants, instead of forming spherical liquid-like droplets, precipitated heavily under all conditions tested (Fig. 4G, Additional file 1: Fig. S5). This finding is consistent with our in vivo results (Fig. 4H). We complemented PQBP1 knockout cells with PQBP1 wild-type protein or mutant proteins, maintaining them at essentially endogenous levels (Additional file 1: Fig. S11A-B). As shown in Fig. 4H and Additional file 1: Fig. S10C-D, the removal of the C-terminus resulted in a decrease in the intracellular condensates of PQBP1, in the case of p.Arg214fs, p.Glu183fs. Moreover, hardly any condensate was observed in cells with the two PQBP1 mutants, p.Arg155* and p.Arg142*. Together, these results demonstrated that in addition to LoF mutations which lower protein levels, loss of phase-separation-prone regions on dosage-sensitive gene products affect their phase separation process.

### Impaired phase separation caused by LoF genetic perturbations causes disturbed phenotypes which can be restored by rescuing phase separation

**Fig. 5:**
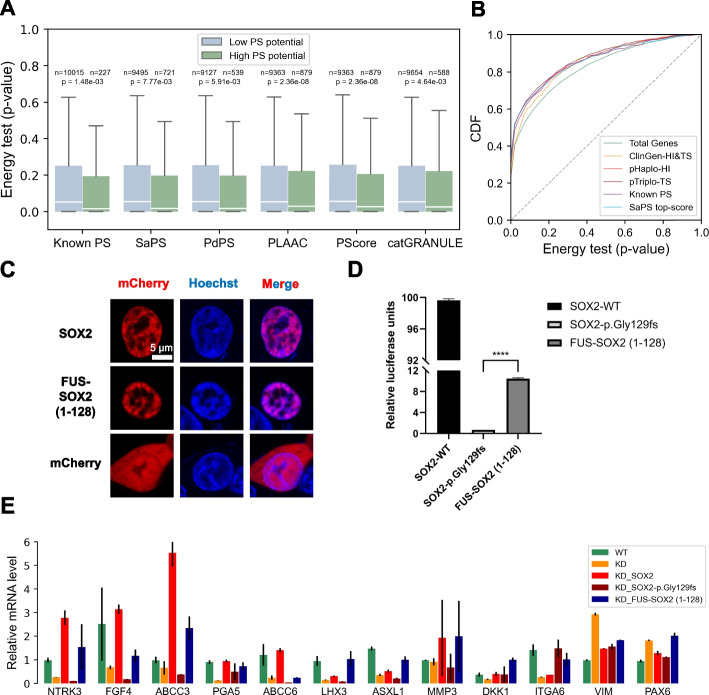
LoF genetic perturbations on phase-separating genes cause disturbed phenotypes. **A** Comparison of *p*-value of energy test between known phase-separating (PS) protein or proteins with top 5% phase separation score and other proteins in the human proteome. *P*-value was calculated with the two-sided Mann–Whitney *U* test. **B** Plot of cumulative distribution function for *p*-value of energy test of gene sets. ClinGen-HI&TS genes: haploinsufficient/triplosensitive gene from ClinGen. pHaplo-HI: haploinsufficient genes according pHaplo score. pTriplo-TS: triplosensitive genes according pTriplo score. SaPS top-score: genes with top 5% SaPS scores. **C** Confocal images of live SOX2 knockdown HEK 293T cells transfected with mCherry-tagged wild-type SOX2 protein, FUS-SOX2 (1–128) proteins and mCherry, and stained with Hoechst (blue). Scale bar, 5 µm. **D** SOX2 knockdown (KD) cells were transfected with the indicated SOX2 constructs. Data are shown as percentage over the SOX2-Full Length transfection. The values shown represent the mean ± SEM, ****P < 0.0001 (*n* = 3 replicates). **E** SOX2 target gene mRNA expression in cell lines

The results thus far demonstrated that dosage-sensitive gene products tend to undergo phase separation and pathogenic variations in dosage-sensitive genes lead to an impaired phase separation process. To evaluate the effects of impaired phase separation on cellular behavior, we utilized a perturb-seq dataset. This dataset provided single-cell RNA-sequencing readouts after CRISPR-based perturbation of gene expression [40]. This genome scale profiling of genetic perturbations enables systematic assignment of cellular phenotypes for each gene perturbation. To test whether perturbation of phase-separating genes results in more dramatic phenotypic changes, we applied an energy test [40] that evaluates global transcriptional changes of each gene perturbation. As shown in Fig. 5A, the *p*-value obtained from this energy test of genes with high phase separation scores were significantly lower than those genes with low phase separation scores. This finding indicated that the LoF of phase-separating genes results in dramatic phenotypic changes when compared to non-phase-separating genes. To further measure the severity of perturbation of phase-separating genes, we compared the *p*-values from energy test of known dosage-sensitive genes with those of phase-separating genes. As shown in Fig. 5B, the *p*-value obtained through the energy test of known dosage-sensitive genes was similar to those of known phase-separating genes, as well as those for high-phase-separation scores. These results demonstrated that LoF genetic perturbations on phase-separating genes cause similar transcriptional phenotypes as those on dosage-sensitive genes, suggesting that perturbation of phase-separating genes results in dosage-sensitive-like effect.

Considering that most TFs obtain phase separation ability, we then attempted to assess whether it could be possible to restore the function of phase-separating TFs carrying LoF mutations by rescuing their phase separation abilities. We first verified whether it is possible to rescue phase separation of SOX2 in the knockdown cell line by ectopically expressing chimeric SOX2 proteins with IDRs promoting phase separation. To this end, we generated FUS-SOX2 (1–128) chimeric proteins by connecting IDR-truncated SOX2 (residues 1–128) to the downstream of FUS IDR (residues 1–214). Previous experiments have characterized FUS IDR to phase separate in vitro [41]. When we complemented SOX2 knockdown cells with SOX2 protein or FUS-SOX2 (1–128) chimeric protein, we observed similar intracellular puncta compared to control cells expressing the mCherry-vector alone (Fig. 5C and Additional file 1: Fig. S10A-B, S11C-E). We next sought to determine whether the transcriptional activity of SOX2 proteins depends on IDR-driven phase separation. We used the dual-luciferase reporter assay to detect SOX2-dependent transcriptional activity. For this study, 26 copies of the canonical SOX2 binding motif are inserted upstream of a promoter for firefly luciferase. By co-transfecting plasmids driving the expression of SOX2 proteins with firefly luciferase plasmids and Renilla luciferase plasmids, significant luciferase activities could be detected in cells (Fig. 5D). Since both SOX2 and FUS-SOX2 (1–128) provide similar phase-separating abilities as shown above, we attempted to rescue the transcription activity of SOX2 with FUS-IDR fused SOX2. Compared with SOX2 full-length proteins, the IDR deficient SOX2-p.Gly129fs proteins significantly reduced transcriptional activity, but the FUS-IDR fused SOX2 (1–128) improved luciferase expression, which indicated FUS-IDR rescued transcriptional activity of IDR-deficient SOX2. In addition, we used RT-qPCR experiments to analyze the effects of rescuing the SOX2 heterozygous knockdown cell line with the chimeric FUS-SOX2 (1–128) on the expression of endogenous target genes. For most of all twelve SOX2 activated target genes in TRRUST database [42], the heterozygous knockdown cells and the heterozygous knockdown cells expressing the IDR-deficient SOX2-p.Gly129fs proteins significantly reduced transcriptional activity compared with wild-type cells, but as with the wild-type SOX2, the FUS-SOX2 (1–128) improved expression of endogenous target genes in heterozygous knockdown cells (Fig. 5E, Additional file 1: Fig. S12). These evidences demonstrated the importance of phase separation for the transcriptional function of SOX2, implying a possible mechanism to restore LoF perturbations by rescuing phase separation abilities.

### Dosage-sensitive scores derived from population genetics data are effectively predictive of phase separation

**Fig. 6:**
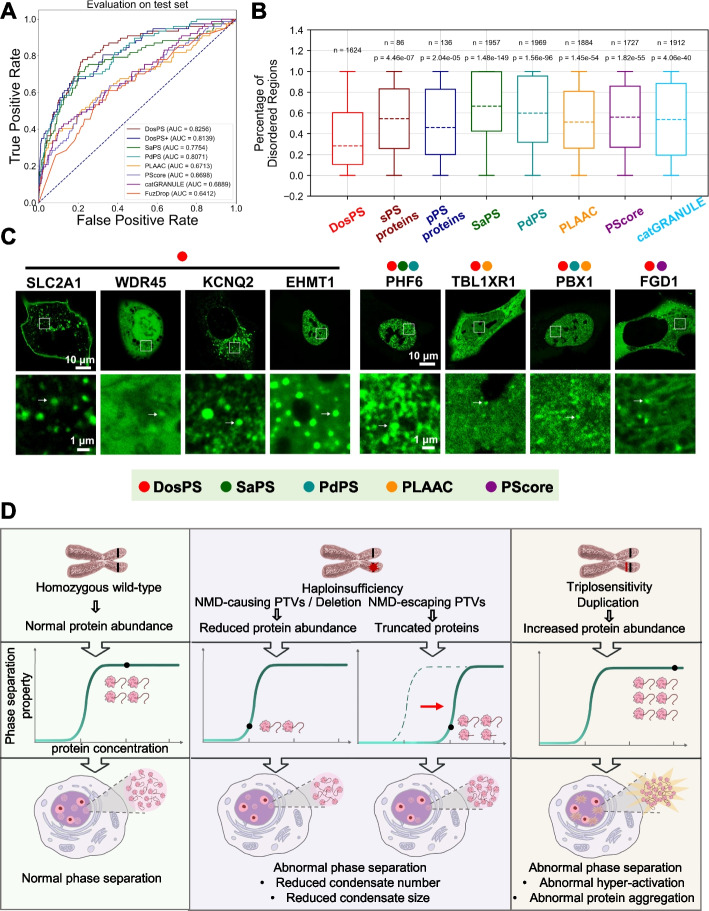
Phase separation predictor based on dosage-sensitive scores. **A** AUC performance of predicting phase-separating proteins on the test set. DosPS + represents LR predictor featuring pLI, LOEUF, pHaplo, pTriplo, SaPS, PdPS, PLAAC, PScore, catGRANULE and FuzDrop scores. **B** Comparison of percentage of disordered regions in proteins sets: self-assembling phase-separating (sPS) proteins, partner-dependent phase-separating (pPS) proteins and proteins with top 10% DosPS, SaPS, PdPS, PLAAC, PScore, catGRANULE score. Disordered regions were calculated with the ESpritz DisProt program. *P*-value was calculated with the two-sided Mann–Whitney *U* test. **C** Representative images of live U-2 OS cells transfected with EGFP tagged top-scored proteins from DosPS predictor. The white arrows indicated the existence of puncta. Scale bars, 10 μm, and 1 μm, respectively. Dots represent the protein belong to top-10%-scored proteins of corresponding predictor. **D** Schematic view of the dosage sensitivity model explained with phase separation

Features of protein sequences and structures that are prone to phase separation have been extensively discussed in previous studies [18]. Nevertheless, available phase separation predictors are far from perfect because of possible neglected principles. The close link between phase separation and dosage sensitivity suggests that phase-separating proteins can be predicted by dosage-sensitive scores derived from population genetics data. To this end, we integrated four dosage-sensitive scores (pLI, LOEUF, pHaplo, and pTriplo) by logistic regression model and established a phase separation predictor called DosPS (dosage sensitivity-based phase separation predictor). As shown in Fig. 6A, the AUC value for DosPS on the test set was 0.8256, which outperformed all currently available phase separation predictors. We also attempted to integrate the dosage-sensitive scores and sequence-based phase separation predictors to improve the prediction performance. However, the integration of sequence-based phase separation predictors such as PLAAC and PScore did not improve the prediction performance of DosPS (Fig. 6A). To demonstrate the differences between DosPS and the other phase separation predictors, we overlapped the top-scored proteins of six predictors (Additional file 1: Fig. S13A). As shown in Fig. 6B, DosPS-top-scored proteins were characterized by a lower percentage of disordered regions, which is different from the preference of other predictors for disordered regions.

To validate the performance of the DosPS predictor, we experimentally validated the top-scored candidates. Candidates included EHMT1, TBL1XR1, SLC2A1, and WDR45 which are specific in DosPS-top-scored proteins and exhibit lower IDR percentage, PHF6, PBX1, KCNQ2, and FGD1 which are included in top-scored proteins of other predictors and exhibit higher IDR percentage. As shown in Fig. 6C, these proteins exhibited appropriate cellular localization and formed prominent puncta in both the nucleus and cytoplasm. These results clearly showed that DosPS constitutes an efficient phase separation predictor featuring dosage-sensitive scores compared to other available tools that solely rely on primary sequence information.

## Discussion

In this study, we established a clear link between dosage sensitivity and phase separation. We showed that products of dosage-sensitive genes possess extremely high phase separation scores. In vitro and in cell experiments further proved that pathogenic variations in dosage-sensitive genes disturb the phase separation process either by reduced protein levels or by loss of phase-separation-prone regions. Multi-omics data analysis further demonstrated that LoF genetic perturbations on phase-separating genes lead to mimic dosage-sensitive effect. Featuring dosage-sensitive scores closely related to phase separation, the novel phase separation predictor DosPS performed better compared to other available tools.

While previous studies explained dosage sensitivity with stoichiometric imbalance [1, 43], we offer a novel theory to explain dosage sensitivity with phase separation. Previous studies failed to consider that among the genes in yeast that are highly sensitive to overexpression, 75% of these genes are not haploinsufficient genes [10]. Another study proposed the dosage-stabilizing hypothesis, stating that dosage-sensitive gene products lose normal function when underexpressed due to insufficient amount of protein, but become toxic when overexpressed due to adverse effects on protein homeostasis or the imbalance of protein composition in complex [10]. However, the molecular mechanisms underlying the dosage-stabilizing hypothesis remained under-researched. Based on our findings that protein products of dosage-sensitive genes are capable of high phase separation, we propose a model to explain dosage sensitivity with concentration-dependent triggering of phase separation process (Fig. 6D). Expression of homozygous wild-type genes generates normal protein levels which are sufficient to trigger the phase separation process. Deletion or NMD-causing mutations result in a defect in phase separation due to reduced protein levels. The loss of phase-separation-prone regions by NMD-escaping mutations reduces the ability of the protein for phase separation, resulting in a phase separation defect. Furthermore, an aberrant increase in gene copy numbers of triplosensitive genes results in over-production of proteins. According to our model, two consequences of such overexpression are possible. First, such aberrant overexpression results in abnormal hyper-activation of related downstream pathways. Alternatively, abnormal accumulation of over-produced protein might result in abnormal aggregation of phase-separating proteins. Previous researches have focused on dosage sensitivity when over-expressing phase-separating proteins, suggesting that excessive aggregation of phase-separating proteins can be toxic to cells [11]. However, in addition to exploring the dosage sensitivity caused by overexpression of phase separation, we also analyzed the dosage sensitivity caused by the under-expression of phase-separating proteins in depth. In addition, we systematically discussed the impact of abnormal phase separation on downstream functions.

In phase separation field, what kind of proteins are prone to phase separate has been extensively discussed [5, 8, 44, 45]. However, previously established phase separation predictors were far from perfect, especially performed poorly for partner-dependent phase-separating proteins. By investigating the relationship between dosage sensitivity and phase separation, we provide a novel approach to predict phase separation. We found that dosage-sensitive scores predicted phase-separating proteins with high confidence for both self-assembling and partner-dependent phase-separating proteins. Since dosage-sensitive scores do not depend on primary sequence information, the partner-dependent phase-separating proteins, which are characterized by a lower percentage in intrinsically disordered regions, are identified by DosPS as well. Consequently, the percentage in disordered regions of high-phase-separation-potential proteins predicted by DosPS is significantly lower. Our analysis strongly suggests that previous sequence-based phase separation predictors are biased toward disordered regions [46]. In comparison, our newly devised approach offers a more reliable avenue for predicting phase-separating proteins, namely by using the dosage-dependent degree of proteins. Our findings provide a novel insight into combining phase separation mechanism and cellular events related to change of protein levels.

While we showed that phase separation represents a potential mechanism of dosage sensitivity, a number of limitations remain to be addressed. Firstly, for the NMD-escaping mutations in haploinsufficient genes which generate truncated proteins with loss of phase-separation-prone regions. Usually, phase-separation-prone regions are not limited to regulating the process of phase separation [47, 48]. For example, key regions for phase separation of SHP2 include the conserved well-folded PTP domain, which acts as a phosphatase regulating the homeostasis of protein tyrosine phosphorylation [47]. Loss of phase-separation-prone regions may disturb functional domains or interaction sites non-relevant to phase separation. Although abnormal phase separation might represent a more general mechanism for dosage sensitivity than currently appreciated theories, other mechanisms may underlie the origin of dosage sensitivity as well. Secondly, the precise relationship between the concentration of protein and the degree of phase separation process needs to be studied in further detail. How to utilize the existing experimental data to predict the threshold concentration of proteins to undergo phase separation in cell is still a challenging task. Lastly, while we show that triplosensitivity is closely linked to phase separation, we did not investigate how overexpression of protein contributes to disease by affecting phase separation. We speculate that overexpression of protein may change the properties of the droplets, such as becoming solid or gel, or cause the continuous activation of biological processes regulated by phase separation resulting in disordered cell states.

## Conclusions

In conclusion, we propose that aberrant phase separation is a biological process associated with the dysfunction of dosage-sensitive genes. We extend the pathogenic mechanism to the abnormal concentration of phase-separating proteins, which closely links the relationship between diseases and phase separation. In the future, we expect that correcting the abnormal phase separation process constitutes a suitable avenue for future treatment of dosage-sensitive diseases.

## Methods

### Data acquisition

Dosage-sensitive information (Dosage Sensitivity Curations, 2021-04-02) was downloaded from Clinical Genome Resource (ClinGen) [20]. LOEUF [22] scores (pLoF Metrics by Gene from gnomAD, 2021-01-05) and pLI [21] scores (Gene constraint scores from ExAC, 2016-02-12) were downloaded from Genome Aggregation Database (gnomAD). List of known phase-separating proteins (2021-06) was downloaded from PhaSepDB [39] (http://db.phasep.pro/). TCGA somatic mutation annotation file (MuTect2 Masked Somatic Mutation, 2021-3-12), RNA-seq data (HTSeq-FPKM, 2021-8-9), copy number variation data (Gene Level Copy Number Scores, 2021-11-29), and TCGA sample clinical information (2021-3-12) were downloaded from TCGA data portal (https://portal.gdc.cancer.gov/). ClinVar [35] vcf mutation data (vcf_GRCh38, 2020-9-14) and mutation summary data (variant_summary.txt, 2020-9-14) were downloaded from the National Center for Biotechnology Information (NCBI). The protein–protein interaction network data were downloaded from a previous study [49].

### Acquisition of the human proteome

The sequence data of the human proteome was downloaded from Uniprot (2020-08-06). The corresponding transcript of each gene in the Ensembl reference library (GRCh38, release-99) was mapped into the canonical proteins in Uniprot by using local blast tool. The parameters were as follows: blastp -outfmt 6 -evalue 1e-5 -num_threads 4. The corresponding protein and transcript were matched with the criteria of 100% match rate and the same protein length.

### Calculating phase separation scores of the human proteome

The SaPS, PdPS, PScore, PLAAC, catGRANULE, and FuzDrop score of each protein was calculated using the corresponding tools under the default parameters [11, 19, 23–25]. PLAAC provides three summary scores for a given sequence, including LLR, CORE, and PRD. Since the LLR score is more appropriate in whole-proteome screening, the normalized LLR score (NLLR) to represent the PLD-forming propensity was used. SaPS and PdPS score based on ten features was used in this study.

### Calculating AUC of predicting phase-separating proteins

Seventy-nine self-assembling human phase-separating proteins identified in a previous study [39] were collected, of which 53 were used for training and 26 were used for independent testing. One hundred twenty-one human partner-dependent phase-separating proteins were collected, of which 70 were used for training and 51 were used for independent testing. In total, 4491 human non-phase-separating proteins were collected, of which 2924 were used for training and 1567 were used for independent testing (Additional file 2: Table S1).

Independent test set was used to evaluate the AUC. Two times the number of proteins compared to phase-separating proteins were randomly selected from the non-phase-separating protein set as negative samples. All self-assembling proteins or partner-dependent phase-separating proteins were selected as positive samples. Above process was repeated 50 times and the mean AUC of scores were calculated respectively for comparison.

### Calculating DM scores of phase separation scores

To account for the confounding effects of factors such as protein half-time on the phase separation scores, the DM values for each gene using rolling medians of phase separation score (PS) were computed:

$$PSDM(i)= PS(i)-rPS(i)$$

where $rPS(i)$ is the rolling median of gene *i* from the scatter plot between confounding factor and phase separation scores. To compute the rolling medians, the following parameters were used: the number of genes in the window is 50 and the number of overlapping genes between adjacent windows is 25.

### Prediction of IDR

The ESpritz DisProt program with the decision threshold set at a 5% false positive rate (FPR) was used to predict potential disordered regions [50].

### Gene set enrichment analysis

Gene set enrichment analysis was applied at webgestalt (http://www.webgestalt.org/) [51] with over-representation analysis (ORA) as method and biological process in Gene Ontology (GO) as pathway database. Genome protein-coding gene set was used as reference set and method of weighted set over was used to reduce redundancy. Enriched pathways were selected based on FDR < 0.05. Gene lists of GO term were downloaded from Gene Ontology (http://geneontology.org/).

### Identifying PTVs in ClinVar database

The vcf mutation data of ClinVar was annotated through SnpEff with reference annotation file in ensemble (release-99) to obtain mutation information (gene, transcription, mutation position, and mutation type). Only mutations in the canonical transcription of each gene were selected. Mutations in ClinVar meeting the following criteria were selected: (1) with the status of pathogenic or likely pathogenic (P/LP); (2) without any conflicting interpretations; (3) with the review status of one or more gold stars. PTVs contained here nonsense, frameshift, and splice-disrupting mutations corresponding to “stop_gained,” “frameshift_variant,” and “splice_region_variant” in annotation of SnpEff.

### Identifying deletion copy number variants in ClinVar

From mutation summary data of ClinVar, “copy number loss” variants meeting the following criteria were identified: (1) with the status of pathogenic or likely pathogenic (P/LP); (2) without any conflicting interpretations; (3) with the review status of one or more gold stars.

### Rules to predict NMD-escaping mutations

The cDNA sequences and positional annotations of exons of each gene were downloaded from Ensembl (GRCh38, release-99). According to the position information of the premature termination codon (PTC) on the cDNA, the following rules were used to predict NMD-escaping mutation [37]: (1) if the PTC is in the last exon; (2) if the PTC is in the last 50 nt of the penultimate exon; (3) if the PTC is < 150 nt away from the start codon; and (4) if the PTC is in a long exon (> 400 nt).

### Quantification of Puncta in cells

CellProfiler 3 was used to quantify the puncta in cells. First, all cells per image were identified based on target protein fluorescence or DAPI fluorescence. All punctas in the cells were subsequently identified under optimal parameters. Indicators including fluorescence intensity and areas for each cell and puncta were finally output by the program. Droplet recognition is implemented by Adapative Otsu’s method, which sets the threshold and divides the image into three parts (foreground, mid-level, and background) according to the brightness. The brightest foreground is the droplet we need. The fluorescence intensity is derived from the sum of the standardized pixel values of the pixels contained in the nucleus or droplets. The normalization method of the pixel value is to scale the metadata of the image so that it is in the range of 0.0–1.0. The average fluorescence intensity is calculated by dividing the fluorescence intensity of the Object by the corresponding number of pixels contained in the object (that is, the area of the Object).

### TruncPS model

#### Dataset of positive phase-separating regions

A number of previous studies selected regions of the phase-separating proteins for verification experiments to obtain the key regions of phase separation. The newly released version of PhaSepDB collects these protein regions selected as experimental region (LLPS regions). Here, repetitive protein regions were removed from PhaSepDB and protein regions that spontaneously phase separate experimentally were manually identified. Finally, 93 positive phase-separating regions in human were obtained (Additional file 5: Table S4).

#### Dataset of negative phase-separating regions

The negative phase-separating regions were derived from two-part proteins. The first part were these remaining regions of phase-separating proteins after removing positive phase-separating regions. Fifty-six regions on phase-separating proteins (region length is greater than 20aa) were obtained. The second part was derived from non-phase-separating proteins. Regions on non-phase-separating proteins were sampled according to the length distribution of the dataset of positive phase-separating regions. The sampling size is twice that of the dataset of positive phase-separating regions. Finally, 242 negative phase-separating regions in human were obtained (Additional file 5: Table S4).

#### Features for the model

Features used by the phase separation predictor SaPS [19] constructed by our laboratory and the embedding feature of the sequence were adopted. The Hydropathy, Kappa, and Net-charge score of a region were calculated by localCIDER using the default parameter [52]. The ESpritz DisProt program with the decision threshold set at a 5% false positive rate (FPR) was used to predict potential disordered regions [50], and the SEG local package with default parameters was used to detect low-complexity domains (LCD) within a given protein sequence [53]. The number of amino acids in the corresponding disordered or low-complexity region divided by the sequence length was defined as the IDR or LCD proportion. Bepler’s model was used to obtain sequence embedding features [54]. The feature vector (amino acid sequence length × 3705) obtained from embedding was averaged in the dimension of amino acid sequence length. Finally, a 3705-dimensional embedding feature vector was obtained.

#### Model training

Our model was constructed with XGBoost model, a tree-based machine learning algorithm with high efficiency and exemplary performance in handling tabular data. The fivefold cross-validation strategy was adopted to test the performance of the XGBoost model on the positive and negative datasets and calculated the average of AUC. At the same time, the mean AUC of PScore score, PLAAC score, IDR proportion, and LCD proportion were calculated respectively for comparison with the XGBoost model. The positive and negative datasets to train the XGBoost model were used to obtain the final model and predicted TruncPS scores of all NMD-escaping mutations.

### DosPS model

A phase separation predictor called DosPS was constructed by utilizing LOEUF, pLI, pHaplo, and pTriplo score using a logistic regression (LR) model. The training set including 53 human self-assembling phase-separating proteins, 70 partner-dependent phase-separating proteins, and 282 randomly sampled non-phase-separating proteins were used to train model. Grid search was used to optimize the “random_state” and “C” parameters of the LR model. The independent test set was used to test the performance of scores. The model trained by training data was used to predict the DosPS score for the human proteome (Additional file 6: Table S5).

### Experiments

#### Cell lines, chemical reagents, and antibodies

HeLa and HEK 293T cell lines were cultured in DMEM with 10% fetal bovine serum and 1% penicillin and streptomycin (Hyclone) at 37 °C and 5% CO_2_. Cell lines were either newly acquired from ATCC or authenticated within 6 months of growth and cells under culture were frequently tested for potential mycoplasma contamination. Lipofectamine 3000 transfection reagent (catalog no.L3000008) and Lipofectamine 2000 transfection reagent (catalog no.11668027) were obtained from Thermo Fisher Scientific. All antibodies used in this study are listed in Additional file 7: Table S6.

#### Cloning of constructs

The full length of Homo sapiens SOX2 (NCBI Entrez Gene ID: 6657), PAX6 (NCBI Entrez Gene ID: 5080), HNRNPK (NCBI Entrez Gene ID: 3190), and PQBP1 (NCBI Entrez Gene ID: 10084) were amplified using PCR from human cDNA, and cloned into the pHis-parallel vector, with a 6 × His tag added at the N-terminus. The mutations in SOX2 and PQBP1 were introduced via PCR and confirmed by DNA sequencing. To generate FUS-SOX2 (1–128) chimaera, human SOX2 (residues 1–128), and FUS-IDR (NCBI Entrez Gene ID: 2521, residues 1–214) sequences were cloned from human cDNA, respectively, and inserted into the pHis-parallel vector with a 6 × His tag. For rescue constructs, PLVX-mCherry-SOX2, PLVX-mCherry-FUS-SOX2 (1–128), PLVX-mCherry-PQBP1, and their mutants were also constructed for expression in cells.

The full length of Homo sapiens EHMT1 (NCBI Entrez Gene ID: 79813), TBL1XR1 (NCBI Entrez Gene ID: 79718), WDR45 (NCBI Entrez Gene ID: 11152), SLC2A1 (NCBI Entrez Gene ID: 6513), PHF6 (NCBI Entrez Gene ID: 84295), PBX1 (NCBI Entrez Gene ID: 5087), KCNQ2 (NCBI Entrez Gene ID: 3784), and FGD1 (NCBI Entrez Gene ID: 2245) were amplified using PCR from human cDNA and cloned into the PLVX-EGFP vector, with a EGFP tag added at the N-terminus.

#### Protein expression and purification

HNRNPK, PQBP1, HNRNPK-∆IDR, PAX6-∆IDR, PQBP1-∆IDR, and PQBP1 mutants were expressed in *E. coli* strain BL21 (DE3) cells. The bacteria were cultured at 37 °C at 220 rpm in a shaker incubator in LB medium to OD600 0.6–0.8, then induced with 0.5 mM IPTG for 16 h at 16 °C. The bacteria were collected by centrifugation at 4000 rpm for 30 min at 4 °C and resuspended in lysis buffer (20 mM Tris–HCl, 200 mM NaCl, 4 M Urea, 0.1 mM PMSF, 1 × protease inhibitor cocktail, pH 7.5) then sonicated for 30 min on ice (180 W, 5 s on and 5 s off). The lysates were collected by centrifugation at 20,000* g* for 40 min at 4 °C. Next, the supernatant was loaded onto Ni^2+^-NTA resin. The column was washed with wash buffer (20 mM Tris–HCl, 200 mM NaCl, 4 M Urea, 30 mM imidazole, pH 7.5). Subsequently, proteins were eluted with elution buffer (20 mM Tris–HCl, 200 mM NaCl, 4 M Urea, 300 mM imidazole, pH 7.5). Eluted proteins were concentrated using Amicon Ultra filters (Millipore) and analyzed by SDS-PAGE.

For SOX2, SOX2-p.Gly129fs, PAX6, HNRNPK-IDR, PAX6-IDR, and PQBP1-IDR protein purification, expression vectors were transformed in *E. coli* strain BL21 (DE3) and cultured at 37 °C to OD600 0.6–0.8, then induced with 0.5 mM IPTG for 4 h at 37 °C. *E. coli* cells were collected by centrifugation at 4000 rpm for 30 min at 4 °C and resuspended in lysis buffer (20 mM Tris–HCl, 200 mM NaCl, 6 M guanidine-HCl, 10 mM β-mercaptoethanol, 0.1 mM PMSF, 1 × protease inhibitor cocktail, pH 7.5) and lysed by sonication for 40 min (180 W, 10 s on and 10 s off). The lysates were clarified by high-speed ultracentrifugation for 40 min at 20,000* g* at 4 °C. The supernatant was purified through Ni^2+^-NTA resin and washed with wash buffer (20 mM Tris–HCl, 200 mM NaCl, 6 M guanidine-HCl, 20 mM imidazole, and 10 mM β-ME, pH 7.5). Protein elution was done with elution buffer (20 mM Tris–HCl, 200 mM NaCl, 6 M guanidine-HCl, 20 mM β-mercaptoethanol, and 300 mM imidazole, pH 7.5). Eluted proteins were concentrated using Amicon Ultra filters (Millipore) and confirmed by SDS-PAGE. PAX6 proteins were then diluted to 20 mL by a low-salt buffer (20 mM Tris–HCl, 100 mM NaCl, 6 M guanidine-HCl, and 10 mM β-mercaptoethanol, pH 8.0), and further purified over a HiTrap™ Q column according to the manufacturer’s protocol (Cytiva). Fractions containing PAX6 proteins were pooled, concentrated, and analyzed by SDS-PAGE.

All proteins were labeled with Alexa Fluor 488 (Thermo Fisher) and all purification steps were performed at 4 °C.

#### Phase-separated droplet formation

Phase-separated droplets of SOX2, SOX2-p.Gly129fs, PQBP1, PAX6, HNRNPK-IDR, PAX6-IDR, PQBP1-IDR, HNRNPK-∆IDR, PAX6-∆IDR, and PQBP1-∆IDR proteins formed by a quick dilution of the purified protein out of denaturing buffer into phase separation buffer containing 25 mM Tris–HCL pH 7.5 and various concentrations of NaCl to reach the final protein concentrations. Comparison of PQBP1 and its mutants was performed in 150 mM NaCl, 20 mM Tris–HCl pH 7.5, with protein concentrations ranging from 5 to 200 μM. Comparison of SOX2 and SOX2-p.Gly129fs were performed in 150 mM NaCl, 20 mM Tris–HCl pH 7.5, and 5 M NaCl pH 7.5, with protein concentrations ranging from 2.5 to 20 μM. Moreover, comparison of PQBP1-IDR and PQBP1-∆IDR was performed in 50 mM NaCl, 20 mM Tris–HCl pH 7.5. Comparison of HNRNPK-IDR and HNRNPK-∆IDR was performed in 150 mM NaCl, 20 mM Tris–HCl pH 7.5. Comparison of PAX6-IDR and PAX6-∆IDR was performed in 3 M NaCl, 20 mM Tris–HCl pH 7.5.

HNRNPK and HNRNPK-∆IDR proteins were dialyzed into a dialysis buffer (25 mM Tris–HCl, 500 mM NaCl, and 0.1 mM PMSF, pH 7.5) at 4 °C overnight, and then concentrated and quickly diluted into a phase separation buffer containing 25 mM Tris–HCl pH 7.5 and different concentrations of NaCl.

All phase diagrams were obtained on 384-well microscopy plates (Cellvis) and incubated at room temperature for 30 min before being imaged on an Olympus SpinSR spinning disk confocal super-resolution microscope with a × 100 oil objective.

#### Fluorescence Recovery After Photobleaching (FRAP) measurements

In vitro FRAP experiments were carried out with a NIKON A1 microscope equipped with a × 100 oil objective. Droplets were bleached with a 488-nm laser pulse (3 repeats). Recovery from photobleaching was recorded for the indicated time.

#### Generation of heterozygous knockdown or knockout cell lines

Knockdown of SOX2 and PAX6 and knockout of PQBP1 were performed using HEK 293T and HeLa cells, respectively. All the small guide RNAs (sgRNA) used in this study were selected using the CRISPR design tool (https://portals.broadinstitute.org/gppx/crispick/public). To generate the SOX2 knockdown (KD) HEK 293T cell line, the sgRNA (target sequence 5′-CGGCAATAGCATGGCGAGCG-3′) targeting the first exon of SOX2 genome was used. Knockout of PQBP1 was conducted in HEK 293T cells with two sgRNAs targeting the second exon of PQBP1 genome (5′-TCGAACACCTTGTACCAGCT-3′ and 5′-TGGTGGTAGGCCCTCCAACC-3′). Knockdown of PAX6 was conducted in HeLa cells with two sgRNAs targeting the first exon of PAX6 genome (5′-CCAGCCAGAGCCAGCATGCA-3′ and 5′-CTGGTCTTTCTGGGACTTCG-3′). Cells were transfected with sgRNAs by using Lipo3000 (Thermo Fisher Scientific) according to the manufacturer’s instructions. Twenty-four hours after the transfection, 200 cells were plated in a 150-mm cell culture plate. After 2 weeks, single-cell colonies were collected by Colony Cylinders. More than 20 colonies were analyzed by Western blot. Potential knockdown or knockout colonies were confirmed by DNA sequencing around the sgRNA targeting site. Mutation results are shown in Additional file 1: Fig. S8D-F.

#### Western blot

Cell lysates were prepared from adherent cells. Proteins were fractionated by SDS-PAGE and transferred to the nitrocellulose filter membranes. The membranes were incubated overnight with primary antibodies at 4 °C, and HRP-conjugated secondary antibodies for 1 h at room temperature. Finally, a chemiluminescence reagent was used to amplify ECL signal and visualize the results. The band intensities were quantified using ImageJ software.

#### Cell immunofluorescence staining

Cells for fluorescence imaging were seeded onto number 1.5 glass bottom dishes, 24 h prior to experiments. Following washing with PBS for 5 min, cells were fixed in 4% (v/v) paraformaldehyde for 15 min and permeabilized with 0.1% (v/v) Triton-X 100 for 15 min. Cells were blocked for 1 h at room temperature with 5% (w/v) BSA containing 1% Tween-20 (PBS-T) in PBS. Cells were sequentially incubated with the indicated primary and secondary antibodies diluted in PBS-T (1:200–1:500) for 1 h. Secondary antibodies were conjugated to either Alexa Fluor 488 or 568. After washing for three times, Pro-Long Gold Antifade reagent (Life Technologies) were mounted onto samples. Imaging was conducted on Nikon A1R HD25 microscope or Olympus SpinSR rotary confocal microscope (100 × oil objective). Thresholds were kept constant across all images for endogenous cell immunofluorescence.

#### Cell culture and transfection

Cells were cultured in Dulbecco’s modified Eagle’s medium (Gibco) supplemented with 10% fetal bovine serum and 1% penicillin and streptomycin (Hyclone) at 37°C and 5% CO_2_. All cell lines tested negative for mycoplasma contamination and grown to ~ 70% confluence for transfection. SOX2 knockdown HEK 293T cells were transfected with PLVX-mCherry-FUS-SOX2 (1–128), PLVX-mCherry-SOX2, and its mutant by using Lipofectamine 3000 (Thermo Fisher Scientific) according to the manufacturer’s instructions. And PQBP1 knockout HEK 293T cells were transfected with PLVX-mCherry-PQBP1 and its mutants. Cells were incubated with transfection mixture for 6–16 h and replaced with fresh medium. Live cell images were acquired using an Olympus SpinSR spinning disk confocal super-resolution microscope with a × 100 oil objective.

#### Quantification of relationship between protein concentration and phase separation ability in vivo

To quantify relationship between protein concentration and phase separation ability in vivo, mCherry tagged PQBP1 were transiently transfected in PQBP1 knockout HEK 293T cells in 35-mm glass bottom dish (Cellvis) and imaged on an Olympus SpinSR spinning disk confocal super-resolution microscope with a × 100 oil objective. CellProfiler 3 was used to quantify the puncta properties and mean fluorescence intensity of protein in cells.

#### Dual luciferase reporter assay

SOX2-KD cells were co-transfected with SOX2 expression plasmids, Firefly luciferase reporter plasmids, and the internal control vector pRL-TK (Renilla) using Lipofectamine 3000 (Thermo Fisher Scientific). Cells were incubated with transfection mixture for 12 h and replaced with fresh medium. Twenty-four hours after transfection, cells were lysed and assayed for luciferase activity using the Dual Luciferase Reporter Assay System (Promega). The data represent one of at least three independent assays. Standard deviations of the mean and Student’s *t* test were analyzed using GraphPad Prism 7. The experiments were repeated at least three times.

#### RT-qPCR

Control and SOX2 knockdown HEK 293T cells were transfected with indicated plasmids for 48 h. Total RNA was purified from cells using Trizol and quantified by Nanodrop. Two micrograms of total RNA was reverse transcribed to cDNA using TransScript® One-Step gDNA Removal and cDNA Synthesis SuperMix (TransGen, AT311-832 02). One microliter of 1:5 cDNA dilution was used for quantitative PCR with PerfectStart® Green qPCR SuperMix (TransGen, AQ601-01) on an ABI QuantStudio6 Real-time PCR system. Three replicates for each target gene were tested in each repeated experiment. The primers used in this experiment are listed in Supplementary table 7. We used α-tubulin to normalize the data and calculated the normalized fold change for each target gene.

### Supplementary Information

**Additional file 1: Figure S1.** Comparation of phase separation score between human proteome and gene products with high haploinsufficiency potential with other phase separation predicters. A-E. Comparison of PLAAC, PdPS, Pscore, catGRANULE and FuzDrop score between the human proteome and haploinsufficient(HI) gene products from ClinGen or top-20%-scored proteins in the human proteome ranked by haploinsufficient measures. *P*-value was calculated with the two-sided Mann–Whitney U test. **Figure S2.** A strong correlation between haploinsufficiency and phase separation with other phase separation predicters. A-F. Kernel density regression plot of LOEUF, pLI, pHaplo socre and SaPS, PdPS, PLAAC, Pscore, catGRANULE and FuzDrop score. The coefficient is represented with the Spearman correlation coefficient. *P*-value was calculated with the Spearman’s rank correlation test. **Figure S3.** Dosage-sensitivity correlated with other factors. A-C. Kernel density regression plot of LOEUF, pLI, pHaplo socre and protein half-life, mRNA half-life and translation rate. The coefficient is represented with the Spearman correlation coefficient. *P*-value was calculated with the Spearman’s rank correlation test. **Figure S4.** Enriched pathways of gene products with high haploinsufficiency potential orphase separation potential. A. The enriched pathways network diagram of gene products with top 5% LOEUF score. The hexagon represents the enriched pathway. The size rank of hexagons represents the size rank of proteins included in each pathway. The dot represents proteins. The red dot represents the known phase-separating proteins in PhaSepDB. B. The enriched pathways network diagram of proteins with top 5% SaPS score. The hexagon represents the enriched pathway. The size rank of hexagons represents the size rank of proteins included in each pathway. The dot represents proteins. The red dot represents the known haploinsufficient gene products in ClinGen. **Figure S5.** Coomassie stained SDS-PAGE gel for the expression of different recombinant proteins. A. SDS-PAGE separation of recombinant PQBP1 protein and its mutants. B. SDS-PAGE separation of recombinant SOX2 protein, SOX2-p.Gly129fs, PAX6 protein and HNRNPK protein. C. SDS-PAGE separation of recombinant HNRNPK-IDR, PAX6-IDR, PQBP1-IDR, HNRNPK-∆IDR, PAX6-∆IDR, and PQBP1-∆IDR. M: molecular weight marker. **Figure S6.** Phase diagrams of protein-IDR or protein-∆IDR. A. Phase diagram of HNRNPK-IDR and HNRNPK-∆IDR with 150 mM NaCl concentration. B. Phase diagram of PAX6-IDR and PAX6-∆IDR with 3 M NaCl concentration. C. Phase diagram of PQBP1-IDR and PQBP1-∆IDR with 50 mM NaCl concentration. Scale bars, 20 µm. **Figure S7.** Circos plot displaying information of mutations in haploinsufficient genes in ClinVar database. A. The histograms represent the number of NMD-causing mutations, deletion CNVs and NMD-escaping mutations in ClinVar database. The yellow lines represent protein-protein interactions. The red genes represent the known phase-separating proteins in PhaSepDB. **Figure S8.** LoF mutations in haploinsufficient genes destabilize condensate activity *in cell. *A-C. Western blot analysis of SOX2 (HEK 293T), PAX6(HeLa) and PQBP1(HEK 293T) protein level in wild-type and knockdown/knockout cells. D-E. Mutation results of heterozygous knockdown or knockout cell lines. G-H. Confocal multiple cells images of endogenous SOX2 or PAX6 in wild-type (WT) cells and knockdown (KD) cells with heterozygous deletion of SOX2 (HEK 293T cells) or PAX6 (HeLa cells). Cells were stained with SOX2 antibody (red), PAX6 antibody (green) and DAPI (blue). Scale bar, 20 μm. I-J. Comparison of sum area of puncta per cell between SOX2/PAX6 wild-type (WT) cells and knockdown (KD) cells. *P*-value was calculated with the two-sided Mann–Whitney U test. **Figure S9.** Predicting phase separation impact score of NMD-escaping mutations with TruncPS. A. Veen diagram among genes with 3 types of rules escaping from NMD mechanism. B. Schematic view of the TruncPS model. C. Distribution of truncation percentage of NMD-escaping mutations in haploinsufficient genes. D. Distribution of TruncPS score of NMD-escaping mutations in haploinsufficient genes. **Figure S10.** Quantification of puncta in cells transfected with different proteins. A. Confocal multiple cell images of live SOX2 knockdown HEK 293T cells transfected with mCherry tagged wild-type SOX2 protein, FUS-SOX2 (1-128) protein, NMD-escaping mutant SOX2 protein and mCherry. Scale bar, 20μm. B. Comparison of fluorescence intensity of puncta per cell, number of puncta per cell and sum area of puncta per cell between live SOX2 knockdown HEK 293T cells transfected with mCherry tagged wild-type SOX2 protein, FUS-SOX2 (1-128) protein, NMD-escaping mutant SOX2 protein and mCherry. C. Confocal multiple cell images of live PQBP1 knockout HEK 293T cells transfected with mCherry tagged wild-type PQBP1 protein, NMD-escaping mutant PQBP1 proteins and mCherry. Scale bar, 20μm. D. Comparison of fluorescence intensity of puncta per cell, number of puncta per cell, sum area of puncta per cell and mean fluorescence intensity per cell between live PQBP1 knockout HEK 293T cells transfected with mCherry tagged wild-type PQBP1 protein, NMD-escaping mutant PQBP1 proteins and mCherry. **Figure S11.** Western blot analysis of SOX2 and PQBP1 protein levels in wild-type and knockdown/knockout cells. A. Western blot analysis of overexpressed mCherry-fused PQBP1 proteins in PQBP1-KO HEK 293T cells and endogenous PQBP1 in wild-type HEK 293T cells. (PQBP1: 34KD, mCherry-PQBP1-WT: 62KD, mCherry-PQBP1-p.Arg214fs: 58KD, mCherry-PQBP1-p.Glu183fs: 54KD, mCherry-PQBP1-p.Arg155*: 50KD, mCherry-PQBP1-p.Arg142*: 48KD). B. Quantitative analysis of PQBP1 protein level. Each value was normalized to β-Tubulin and converted to the relative expression level of endogenous PQBP1 in HEK 293T. C. Western blot analysis of overexpressed mCherry-fused SOX2 proteins in SOX2-KD HEK 293T cells and endogenous SOX2 in wild-type HEK 293T cells. (SOX2: 34KD, mCherry-SOX2-WT: 62KD, mCherry-SOX2-p.Gly129fs: 44KD, mCherry-FUS-SOX2 (1-128): 65KD). D. Western blot analysis of wild-type HEK 293T cells transfected with 1 ng, 5 ng and 10 ng plasmids of mCherry fused wild-type SOX2 proteins, SOX2-p.Gly129fs proteins and FUS-SOX2 (1-128) proteins. E. Confocal cell images of SOX2-KD HEK 293T cells transfected with 5 ng and 50 ng plasmids of mCherry fused wild-type SOX2 protein, SOX2-p.Gly129fs proteins and FUS-SOX2 (1-128) proteins. Scale bar, 10µm. **Figure S12.** SOX2 target gene mRNA expression in cell lines (Batch 2). **Figure S13.** Description of proteins with high DosPS score. A. Description of overlapping proteins of proteins with top 10% score in the human proteome for phase separation predictors. B. The enriched pathways network diagram of DosPS-top-10%-scored proteins. The hexagon represents the enrichment pathway. The size rank of hexagons represents the size rank of proteins included in each pathway. The red dot represents the known phase-separating proteins in PhaSepDB. The gray dot represents the proteins related with membraneless organelles in PhaSepDB.

**Additional file 2: Table S1.** Dataset of human phase-separating proteins and non-phase-separating proteins. The list contains proteins used to calculate AUC of predicting phase-separating proteins.

**Additional file 3: Table S2.** LoF mutations in haploinsufficient genes in ClinVar database.

**Additional file 4: Table S3.** Mutaion information in experiments.

**Additional file 5: Table S4.** Dataset of human phase-separating regions and non-phase-separating regions.

**Additional file 6: Table S5.** DosPS score of proteins in the human proteome.

**Additional file 7: Table S6.** Antibodies that were used in this study.

**Additional file 8: Table S7.** Primer sequences used in the experiment

**Additional file 9.** Review history.

## Data Availability

All study data are included in the article and supporting information.
