# Supplementary material for: Phase separation as a possible mechanism for dosage sensitivity
Source: Genome Biol. 2024 Jan 15;25:17. doi: 10.1186/s13059-023-03128-z (PMC10789095; doi:10.1186/s13059-023-03128-z)
Supplement: Supplementary file 1 — Additional file 1: Figure S1. Comparation of phase separation score between human proteome and gene products with high haploinsufficiency potential with other phase separation predicters. A-E. Comparison of PLAAC, PdPS, Pscore, catGRANULE and FuzDrop score between the human proteome and haploinsufficient(HI) gene products from ClinGen or top-20%-scored proteins in the human proteome ranked by haploinsufficient measures. P-value was calculated with the two-sided Mann–Whitney U test. Figure S2. A strong correlation between haploinsufficiency and phase separation with other phase separation predicters. A-F. Kernel density regression plot of LOEUF, pLI, pHaplo socre and SaPS, PdPS, PLAAC, Pscore, catGRANULE and FuzDrop score. The coefficient is represented with the Spearman correlation coefficient. P-value was calculated with the Spearman’s rank correlation test. Figure S3. Dosage-sensitivity correlated with other factors. A-C. Kernel density regression plot of LOEUF, pLI, pHaplo socre and protein half-life, mRNA half-life and translation rate. The coefficient is represented with the Spearman correlation coefficient. P-value was calculated with the Spearman’s rank correlation test. Figure S4. Enriched pathways of gene products with high haploinsufficiency potential orphase separation potential. A. The enriched pathways network diagram of gene products with top 5% LOEUF score. The hexagon represents the enriched pathway. The size rank of hexagons represents the size rank of proteins included in each pathway. The dot represents proteins. The red dot represents the known phase-separating proteins in PhaSepDB. B. The enriched pathways network diagram of proteins with top 5% SaPS score. The hexagon represents the enriched pathway. The size rank of hexagons represents the size rank of proteins included in each pathway. The dot represents proteins. The red dot represents the known haploinsufficient gene products in ClinGen. Figure S5. Coomassie stained SDS-PAGE gel for the exp [file 13059_2023_3128_MOESM1_ESM.pdf]

Figure S1

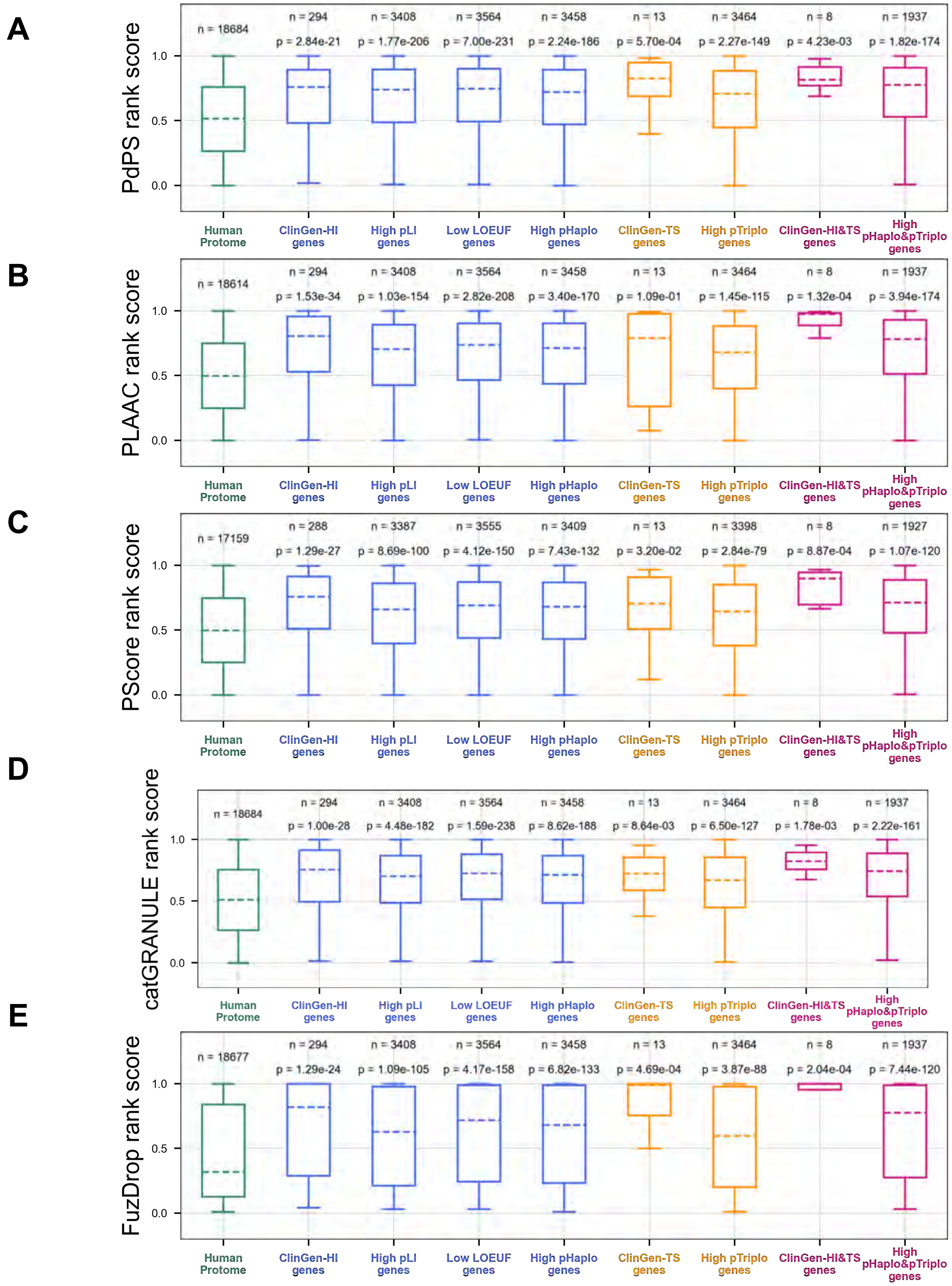

Figure S1. Comparation of phase separation score between human proteome and gene products with high haploinsufficiency or triplosensitivity potential with other phase separation predictors

A-E. Comparison of PLAAC, PdPS, Pscore, catGRANULE and FuzDrop score between the human proteome and haploinsufficient(HI) or triplosensitive(TS) gene products from ClinGen or top-20%-scored proteins in the human proteome ranked by haploinsufficient or triplosensitive scores. P-value was calculated with the two-sided Mann–Whitney U test.

Figure S2

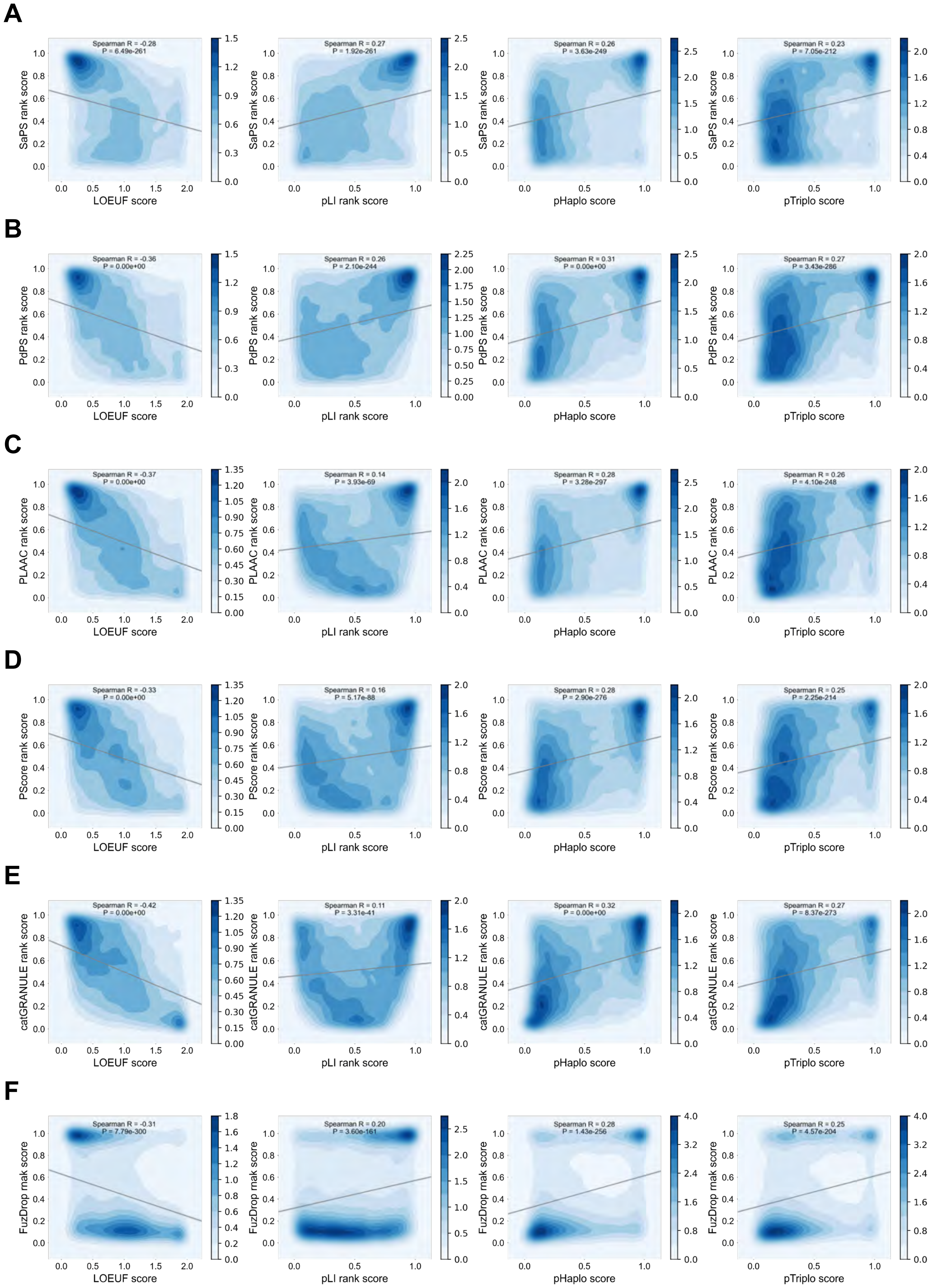

Figure S2. A strong correlation between dosage sensitivity and phase separation with other phase separation predictors

A-F. Kernel density regression plot of LOEUF, pLI, pHaplo, pTriplo socre and SaPS, PdPS, PLAAC, Pscore, catGRANULE and FuzDrop score. The coefficient is represented with the Spearman correlation coefficient. P-value was calculated with the Spearman's rank correlation test.

Figure S3

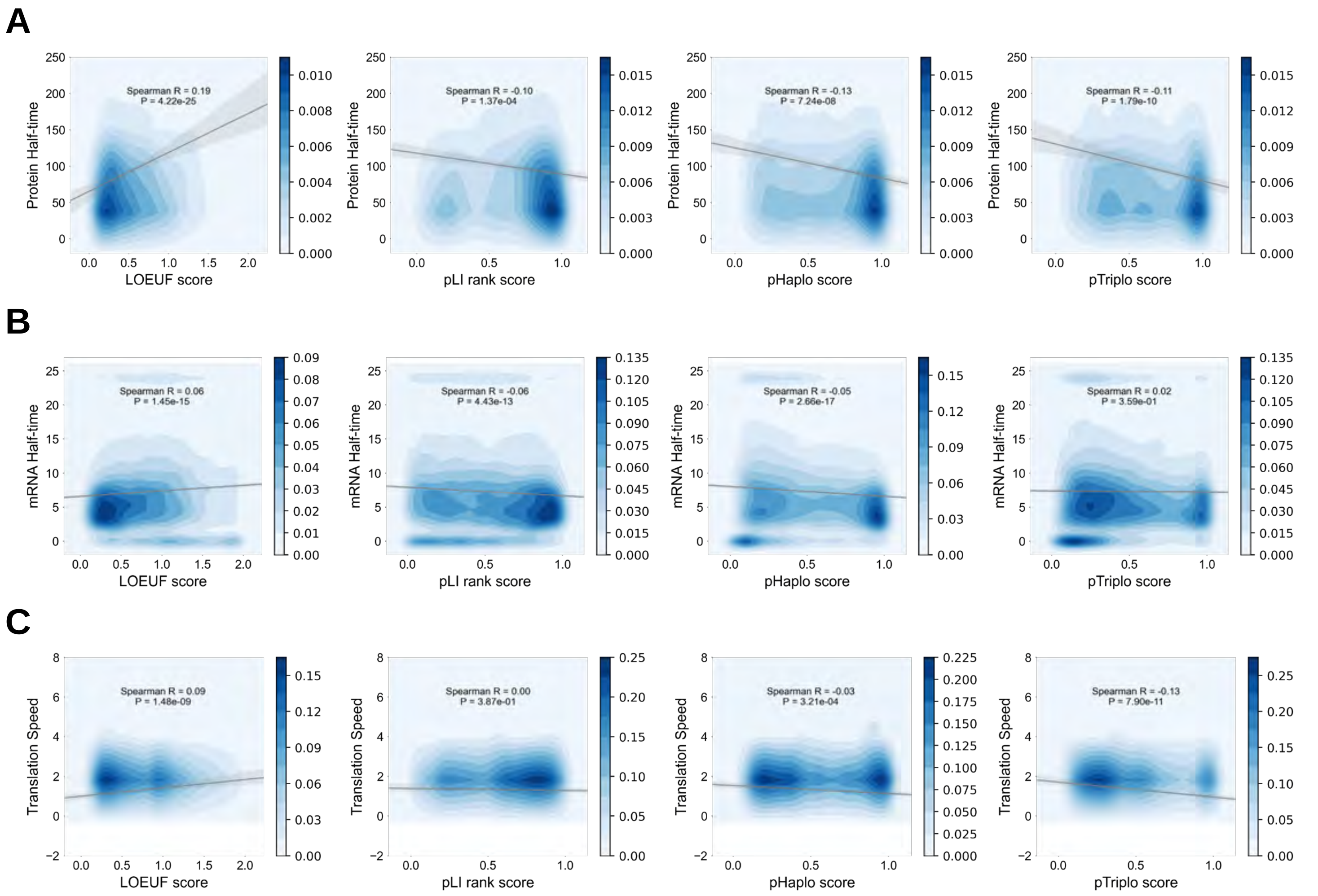

Figure S3. Dosage-sensitivity correlated with other factors

A-C. Kernel density regression plot of LOEUF, pLI, pHaplo socre and protein half-life, mRNA half-life and translation rate. The coefficient is represented with the Spearman correlation coefficient. P-value was calculated with the Spearman's rank correlation test.

Figure S4

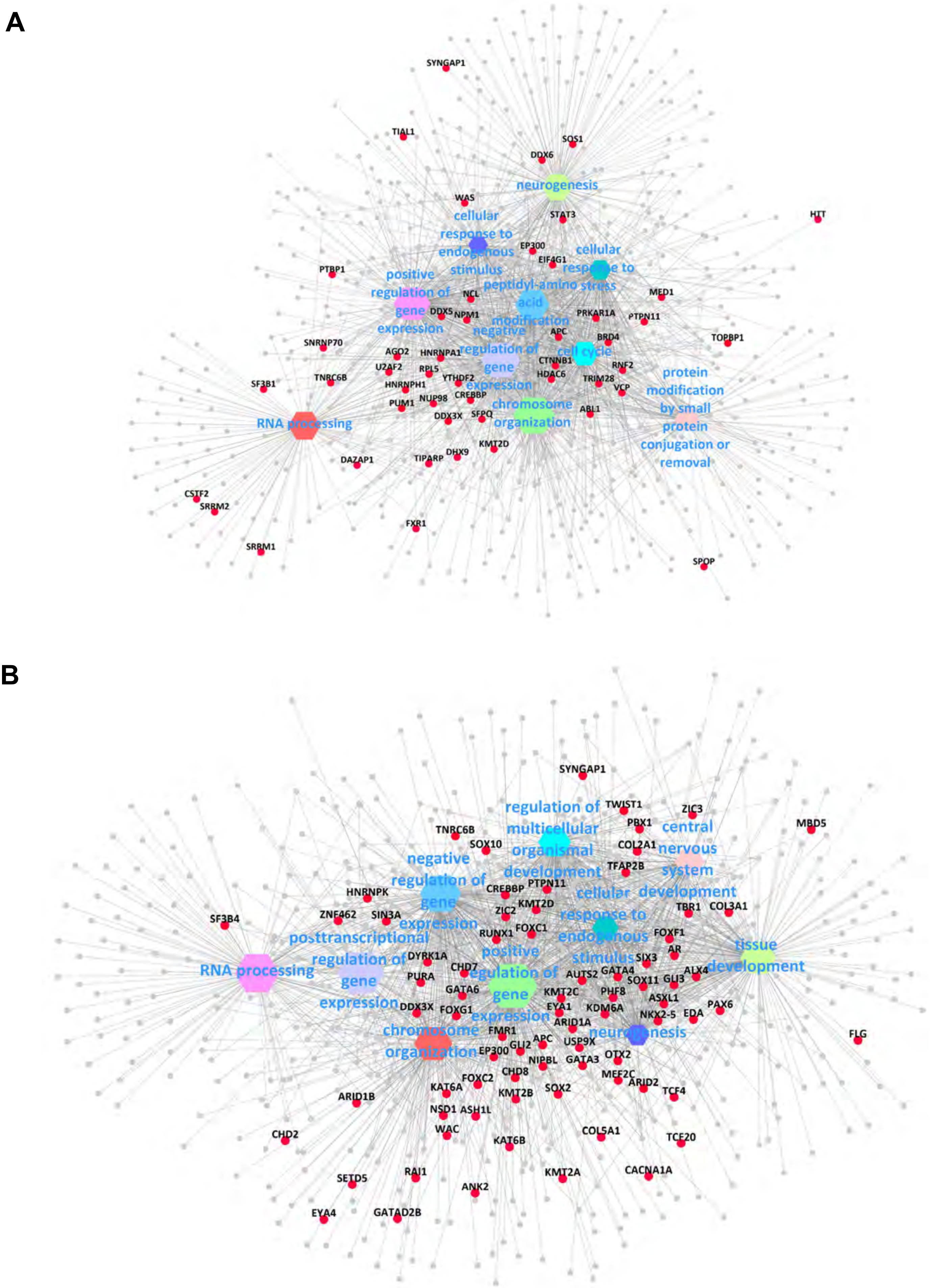

Figure S4. Enriched pathways of gene products with high haploinsufficiency potential or phase separation potential

A. The enriched pathways network diagram of gene products with top 5% LOEUF score. The hexagon represents the enriched pathway. The size rank of hexagons represents the size rank of proteins included in each pathway. The dot represents proteins. The red dot represents the known phase-separating proteins in PhaSepDB. **B**. The enriched pathways network diagram of proteins with top 5% SaPS score. The hexagon represents the enriched pathway. The size rank of hexagons represents the size rank of proteins included in each pathway. The dot represents proteins. The red dot represents the known haploinsufficient gene products in ClinGen.

Figure S5

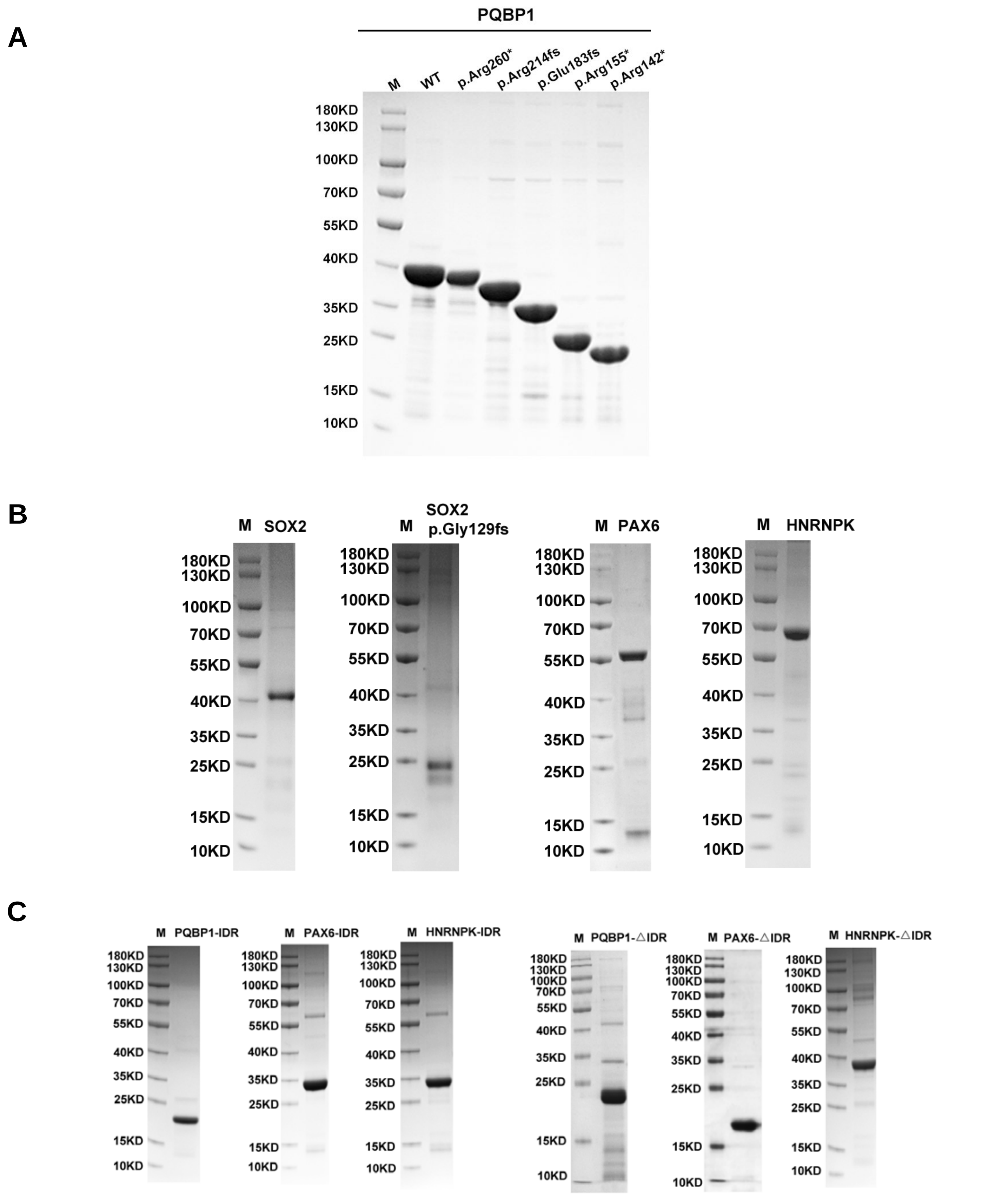

Figure S5. Coomassie stained SDS-PAGE gel for the expression of different recombinant proteins

A. SDS-PAGE separation of recombinant PQBP1 protein and its mutants. **B**. SDS-PAGE separation of recombinant SOX2 protein, SOX2-p.Gly129fs, PAX6 protein and HNRNPK protein. **C**. SDS-PAGE separation of recombinant HNRNPK-IDR, PAX6-IDR, PQBP1-IDR, HNRNPK-△I DR, PAX6-△IDR, and PQBP1-△IDR. M: molecular weight marker.

Figure S6

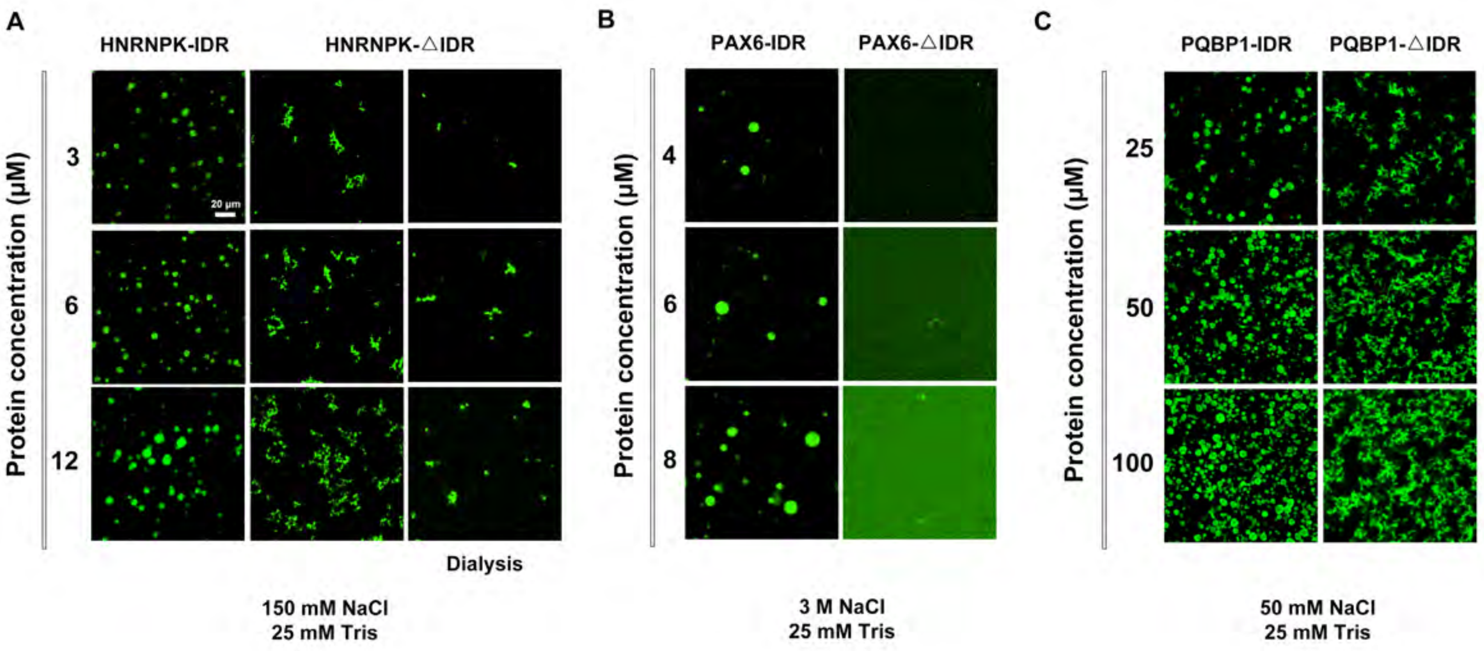

Figure S6. Phase diagrams of protein-IDR or protein-△I DR

A. Phase diagram of HNRNPK-IDR and HNRNPK -△I DR with 150 mM NaCl concentration. **B**. Phase diagram of PAX6- IDR and PAX6-△I DR with 3 M NaCl concentration. **C.** Phase diagram of PQBP1- IDR and PQBP1-△I DR with 50 mM NaCl concentration. Scale bars, 20 μm.

Figure S7

A

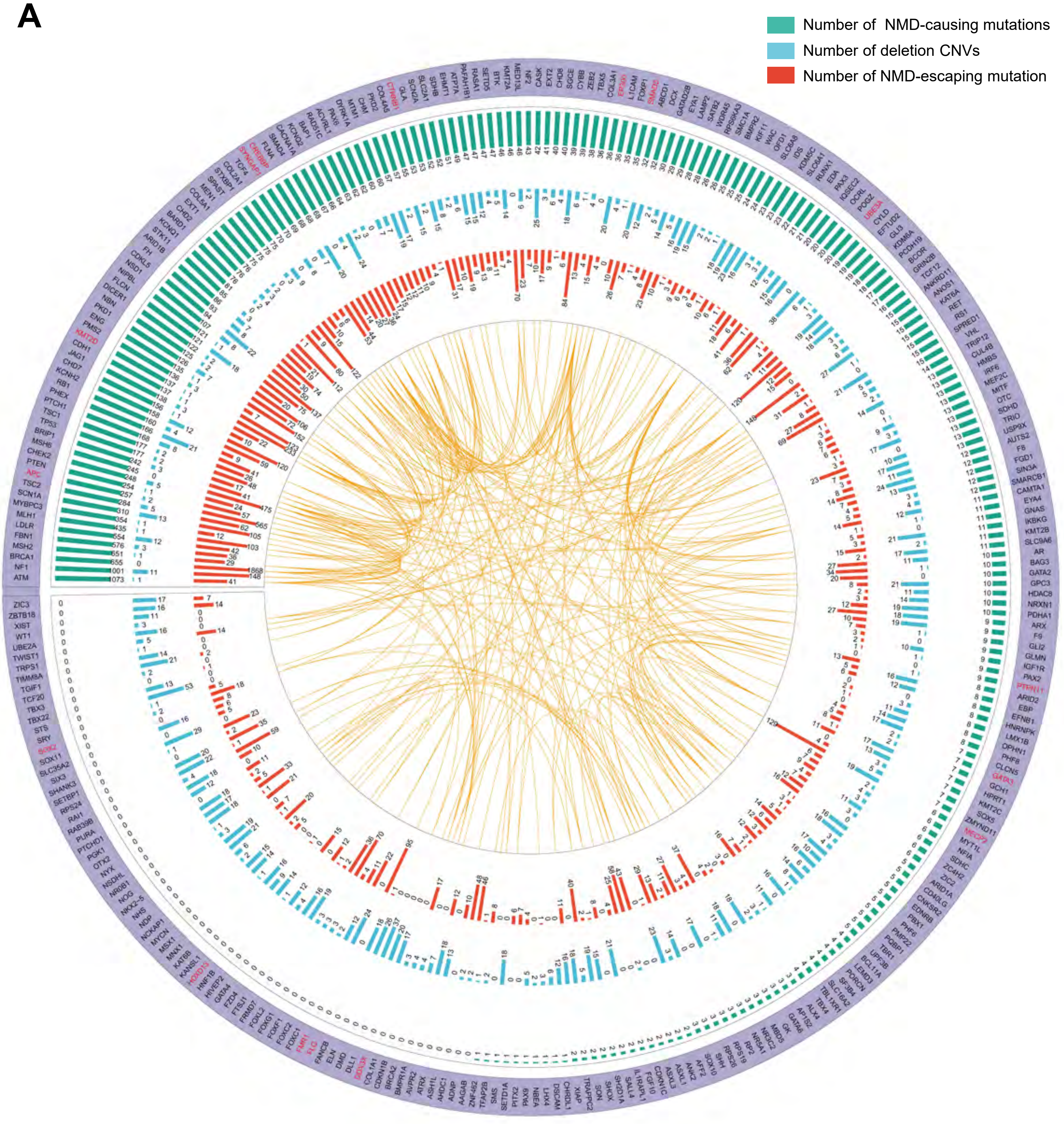

Figure S7. Circos plot displaying information of mutations in haploinsufficient genes in ClinVar database

A. The histograms represent the number of NMD-causing mutations, deletion CNVs and NMD-escaping mutations in ClinVar database. The yellow lines represent protein-protein interactions. The red genes represent the known phase-separating proteins in PhaSepDB.

Figure S8

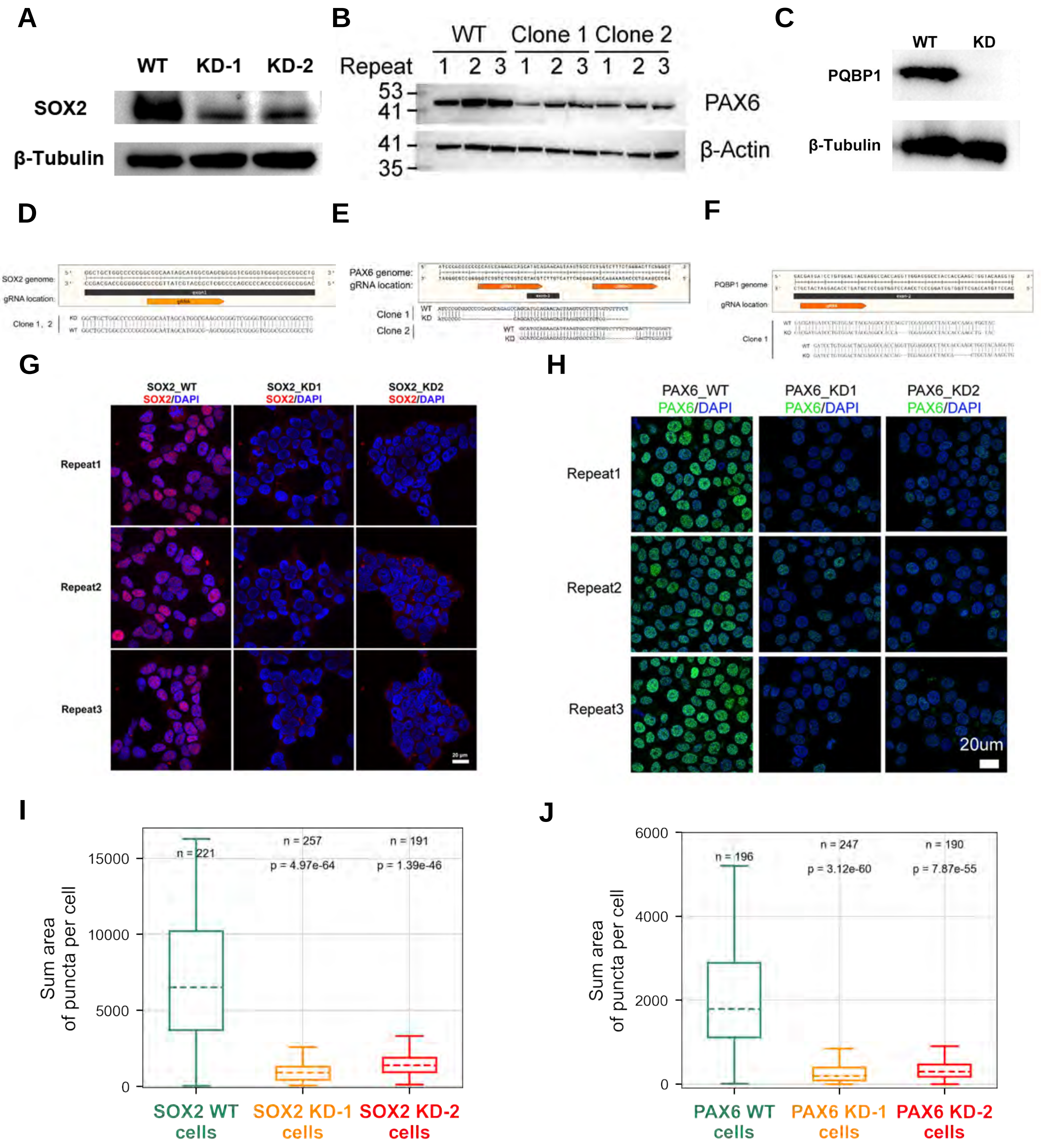

Figure S8. LoF mutations in haploinsufficient genes destabilize condensate activity *in cell*

A-C. Western blot analysis of SOX2 (HEK 293T), PAX6(HeLa) and PQBP1(HEK 293T) protein level in wild-type and knockdown/knockout cells. **D-E**. Mutation results of heterozygous knockdown or knockout cell lines. **G-H.** Confocal multiple cells images of endogenous SOX2 or PAX6 in wild-type (WT) cells and knockdown (KD) cells with heterozygous deletion of SOX2 (HEK 293T cells) or PAX6 (HeLa cells). Cells were stained with SOX2 antibody (red), PAX6 antibody (green) and DAPI (blue). Scale bar, 20 μm. **I-J**. Comparison of sum area of puncta per cell between SOX2/PAX6 wild-type (WT) cells and knockdown (KD) cells. P-value was calculated with the two-sided Mann–Whitney U test.

Figure S9

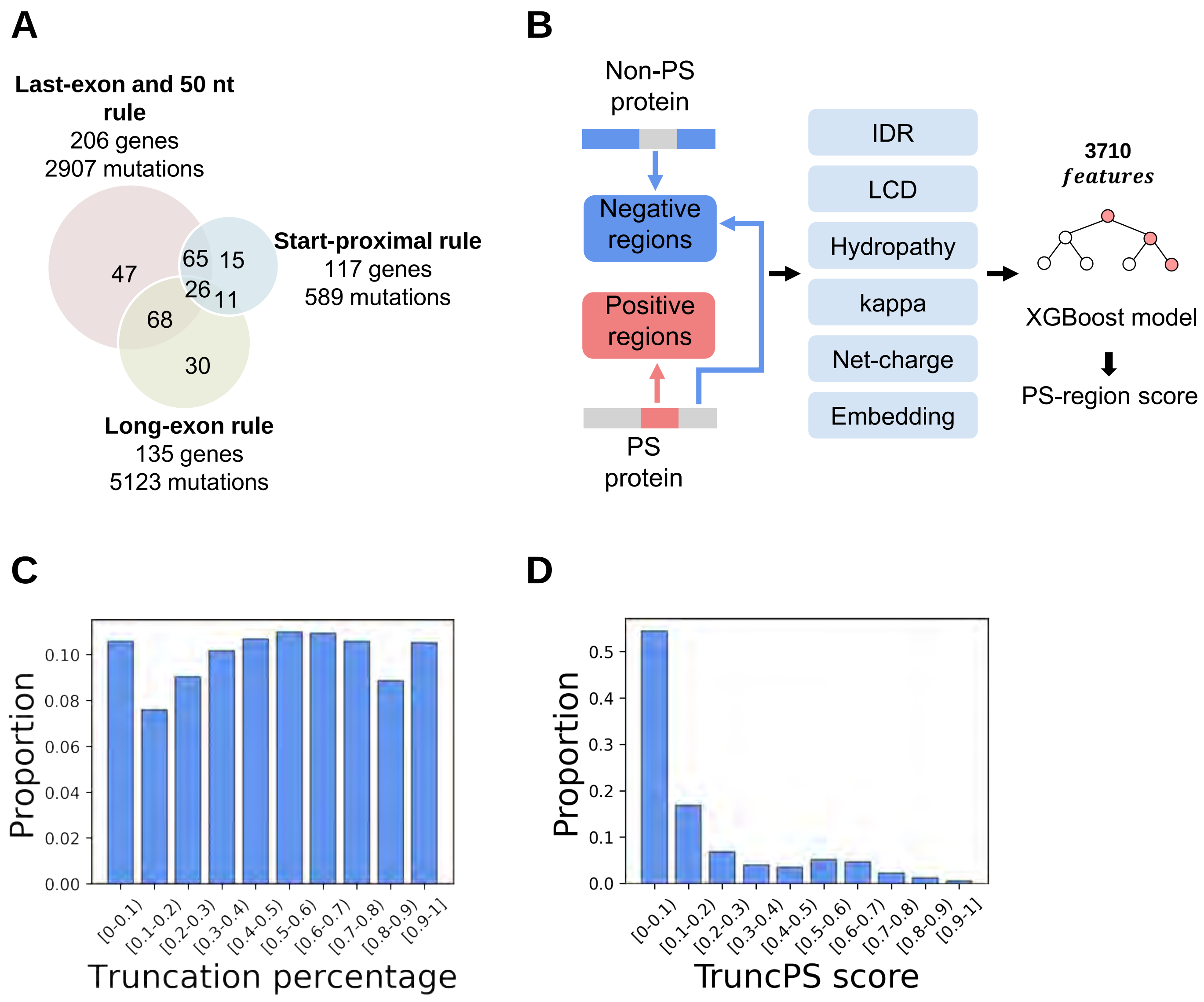

Figure S9. Predicting phase separation impact score of NMD-escaping mutations with TruncPS

A. Veen diagram among genes with 3 types of rules escaping from NMD mechanism. **B**. Schematic view of the TruncPS model. **C**. Distribution of truncation percentage of NMD-escaping mutations in haploinsufficient genes. **D**. Distribution of TruncPS score of NMD-escaping mutations in haploinsufficient genes.

Figure S10

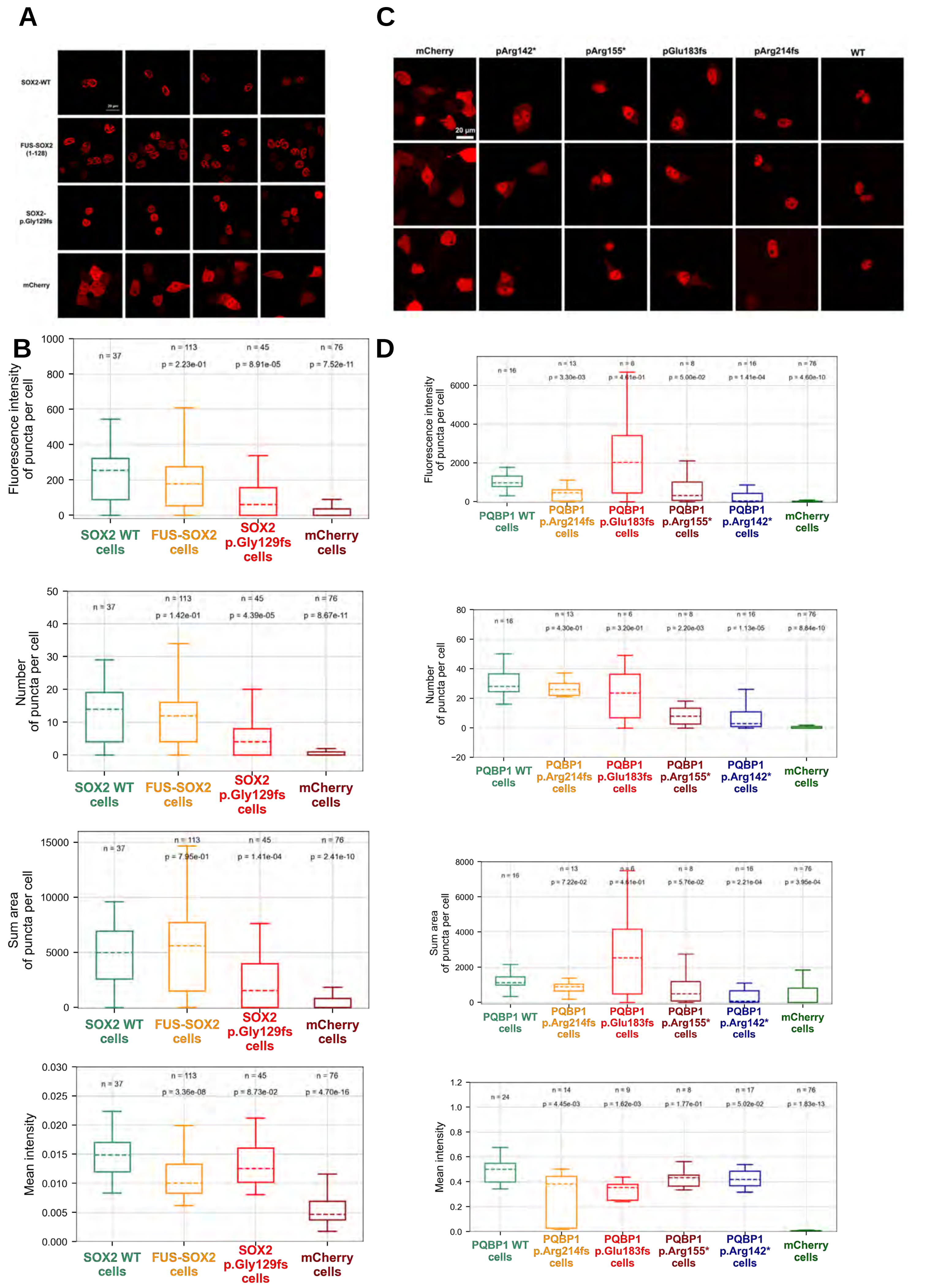

Figure S10. Quantification of puncta in cells transfected with different proteins

A. Confocal multiple cell images of live SOX2 knockdown HEK 293T cells transfected with mCherry tagged wild-type SOX2 protein, FUS-SOX2 (1-128) protein, NMD-escaping mutant SOX2 protein and mCherry. Scale bar, 20μm. **B**. Comparison of fluorescence intensity of puncta per cell, number of puncta per cell and sum area of puncta per cell between live SOX2 knockdown HEK 293T cells transfected with mCherry tagged wild-type SOX2 protein, FUS-SOX2 (1-128) protein, NMD-escaping mutant SOX2 protein and mCherry. **C**. Confocal multiple cell images of live PQBP1 knockout HEK 293T cells transfected with mCherry tagged wild-type PQBP1 protein, NMD-escaping mutant PQBP1 proteins and mCherry. Scale bar, 20μm. **D**. Comparison of fluorescence intensity of puncta per cell, number of puncta per cell, sum area of puncta per cell and mean fluorescence intensity per cell between live PQBP1 knockout HEK 293T cells transfected with mCherry tagged wild-type PQBP1 protein, NMD-escaping mutant PQBP1 proteins and mCherry.

Figure S11

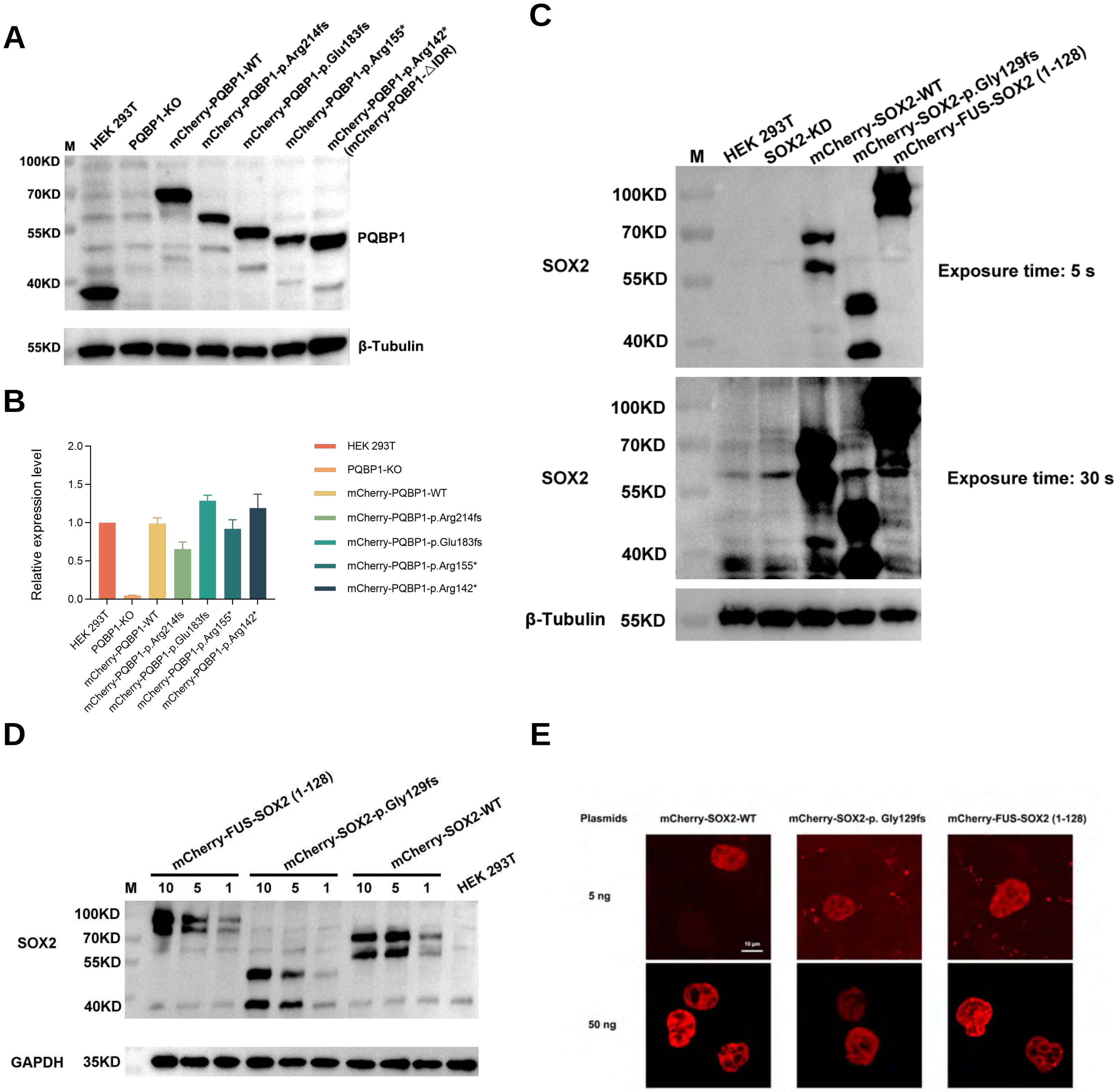

Figure S11. Western blot analysis of SOX2 and PQBP1 protein levels in wild-type and knockdown/knockout cells.

A. Western blot analysis of overexpressed mCherry-fused PQBP1 proteins in PQBP1-KO HEK 293T cells and endogenous PQBP1 in wild-type HEK 293T cells. (PQBP1: 34KD, mCherry-PQBP1-WT: 62KD, mCherry-PQBP1-p.Arg214fs: 58KD, mCherry-PQBP1-p.Glu183fs: 54KD, mCherry-PQBP1-p.Arg155*: 50KD, mCherry-PQBP1-p.Arg142*: 48KD). **B**. Quantitative analysis of PQBP1 protein level. Each value was normalized to β-Tubulin and converted to the relative expression level of endogenous PQBP1 in HEK 293T. **C**. Western blot analysis of overexpressed mCherry-fused SOX2 proteins in SOX2-KD HEK 293T cells and endogenous SOX2 in wild-type HEK 293T cells. (SOX2: 34KD, mCherry-SOX2-WT: 62KD, mCherry-SOX2-p.Gly129fs: 44KD, mCherry-FUS-SOX2(1-128): 65KD). **D.** Western blot analysis of wild-type HEK 293T cells transfected with 1 ng, 5 ng and 10 ng plasmids of mCherry fused FUS-SOX2 (1-128) proteins, SOX2-p.Gly129fs proteins and wild-type SOX2 proteins. **E.** Confocal cell images of SOX2-KD HEK 293T cells transfected with 5 ng and 50 ng plasmids of mCherry fused wild-type SOX2 protein, SOX2-p.Gly129fs proteins and FUS-SOX2 (1-128) proteins. Scale bar, 10μm.

Figure S12

A

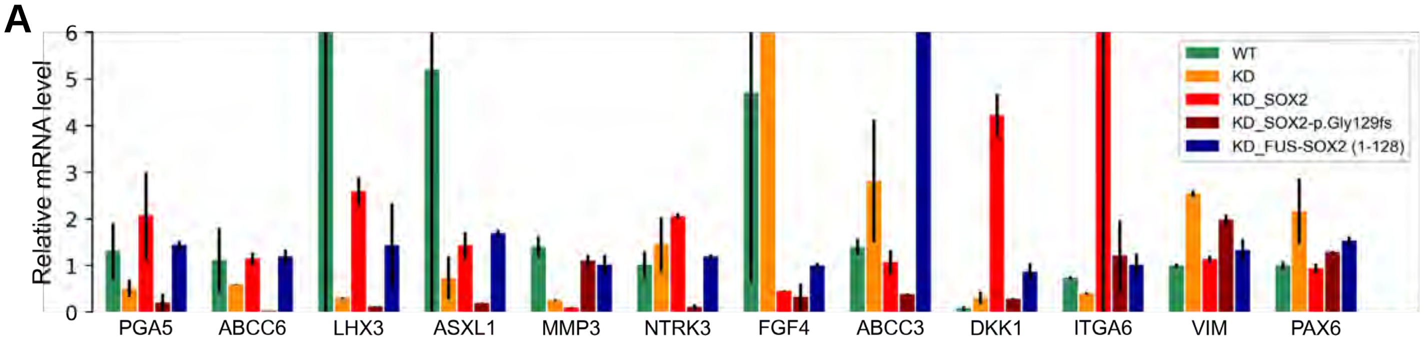

Figure S12. SOX2 target gene mRNA expression in cell lines (Batch 2)

Figure S13

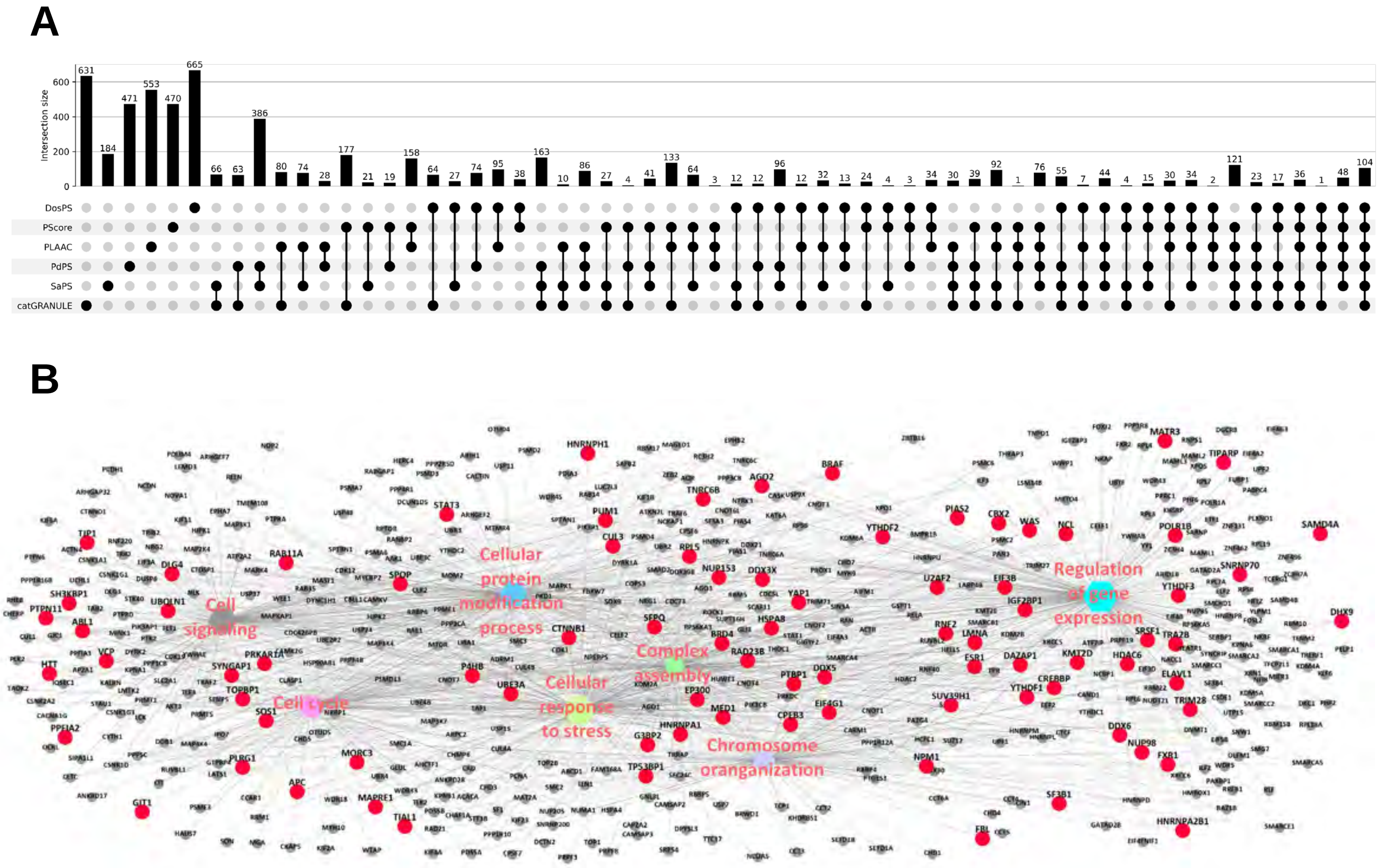

Figure S13. Description of proteins with high DosPS score

A. Description of overlapping proteins of proteins with top 10% score in the human proteome for phase separation predictors. **B.** The enriched pathways network diagram of DosPS-top-10%-scored proteins. The hexagon represents the enrichment pathway. The size rank of hexagons represents the size rank of proteins included in each pathway. The red dot represents the known phase-separating proteins in PhaSepDB. The gray dot represents the proteins related with membraneless organelles in PhaSepDB.
