# Supplementary material for: Phase separation as a possible mechanism for dosage sensitivity
Source: Genome Biol. 2024 Jan 15;25:17. doi: 10.1186/s13059-023-03128-z (PMC10789095; doi:10.1186/s13059-023-03128-z)
Supplement: Supplementary file 9 — Additional file 9. Review history. [file 13059_2023_3128_MOESM9_ESM.docx]

**1^st^ round**

**Reviewer 1**

In this work Liang Yanga and collaborators investigated the relationship between gene dosage sensitivity and phase separation. The proposed hypothesis relies on the observation that upon reaching a certain critical concentration IDP tend to phase separate, forming biomolecular condensates that functionally compartmentalize biological function. However, the author did not consider two relevant parameters: the Csat varies from protein to protein, thus indicating that there is no clear evidence for which the decrease of the 50% of the protein level (for the haploinsufficiency) is sufficient to cross the critical threshold, thus affecting LLPS within cells. The other aspect relates to the fact that while the haploinsufficiency can globally reduce the protein abundance, the local protein concentration within the condensates may remain stable, arguing against a sensitivity to gene dosage. These aspects are not investigated nor discussed, thus limiting the relevance of the findings. We encourage the authors to properly address the contribution of the cellular microenvironments to the dynamic formation of condensates, related to the analyzed proteins.
Although the proposed relationship is of relevance and the combined analytical strategy indeed suggest a possible correlation between sensitivity to gene dosage and the propensity to phase separate of the gene products, the conclusions are not always fully supported by the presented data. Indeed, there are important technical limitations that reduce the impact and the robustness of this study that needed to be addressed before considering the manuscript ready for publications.
.
Mayor criticisms:

1. The droplet assays showed some relevant technical limitations, as they have been performed starting from desaturated recombinant proteins, whose re-folding/structure have not been measured. The obtained results could be strongly affected by the multiple protein conformations that the recProtein could have explored upon resuspension in the Droplet assay buffer. If this point can not be experimentally addressed, it would be necessary to repeat all the droplet assays using native recombinant proteins or properly re-folded one.
2. For the droplet assay, once they will have address the previous comment, the authors should be careful in arguing of complex phase transition (glass- or solid-like states) without providing direct measurements of these specific states. In addition, they should motivate the reason why they are using such a broad range of protein concentrations. All the data lack any quantification and replicates, which strongly limits the relevance of these observations. Finally, the author should have included controls: they need to provide evidence that the observed behavior depends on the predicted IDR of each of the analyzed protein.
3. Related to Figure 3, the author performed genome editing with CRISPR/Cas9, but details are missing both tin the results and methods section. The talk about "knockdown" of SOX2 and PAX6 but this terminology does not mean much related to genome editing: either they generated KO cell lines or haploinsufficiency by targeting one of the two allele. They should provide infos on the kind of genetic perturbation they retrieved, the kind of mutations, using at least two independent cell line/gene to then perform the downstream analyses. For what concerns the IF measurements, it is not specified the numerosity of the analyses samples nor the number of biological replicates by which the used statistical test are not meaningful. Furthermore, if they are comparing WT vs haplo-insufficient cells, how do they justify that they measured a non-linear decrease of the IF intensity? The are no information about the normalization approach used for quantifying the IF intensity, please provide it.
4. The analyses of the relationship between mCherry-PQBP1 levels and the formation of droplet is of interest. However, the experiment misses relevant controls: what is the level of the mCherry-PQBP1, with respect to the endogenous level of PQBP1? The author should provide the same analyses using a PQBP1 lacking its IDR or relevant AA involved in LLPS (that can be determined using the in vitro droplet assay). Please address these points.
5. Related to the results presented in Figure 4D: the protein level of the WT or p.Gly129fs mCherry-SOX2 should be included to address whether the observed differences are related to the protein abundance. Once again, please compare these protein levels with respect to the endogenous SOX2 and provide quantitative values (with replicates) to strengthen the robustness of these data. Same issue for results presented in Figure 4G/H.
6. Referring to Figure 5, the authors attempted to evaluate the effects of altered LLPS on cellular phenotype, namely changes in the gene expression pattern of downstream targets of TFs harboring IDRs (predicted to go through LLPS). The computational prediction of the TF-regulated genes is not appropriate as the used criteria have not been verified/tested by any means. Indeed, selecting the top 50 differentially expressed genes by using the mean expression of cancer datasets retrieved from patients not carrying the PTVs for the analyzed TFs is rather an erroneous assumption, as it would imply that the difference in the gene expression pattern of these group of genes will solely depends on the alteration of the in silico predicted TFs. It is well known that cancers result by the combination of multiple genetic, epigenetic and environmental alterations and assuming the differences are depending on a single mutation of a TFs is simply incorrect. In addition, the author did not provide any supportive data indicating the robustness of the in silico methodology that has been adapted. Therefore, all the obtained analyses do not support the raised conclusions.
7. Referring to the attempt of experimentally demonstrate that the IDRs of TFs is necessary for their biological function, the author analyzed the puncta distribution and the gene expression activity of a reconstituted SOX2-FUS chimeric protein (Figure 5 F-G). However, the obtained data argue against their conclusion as the luciferase assay showed clearly that the chimeric TF is 10 time less efficient in inducing gene expression. Although this result is not per se surprising, it does not argue in favors of the author conclusion, which should be further supported by analyzing the effects of rescuing the SOX2 KD with the chimeric protein (with respect to the WT one) on the expression of endogenous SOX2-targets. In addition, is the chromatin binding and the residence time of its endogenous target maintained? Many controls are missing to reach such a conclusion.

**Reviewer 2**

The manuscript by Dr Li and colleagues addresses an important problem and aims to decipher the molecular basis of dosage sensitivity using phase separation as a possible
mechanism. This problem has been addressed before in PMID 27320918 (ref 11), where the link between concentration dependent cellular toxicity and liquid-liquid phase
separation (LLPS) has been suggested.
Although the connection between dosage sensitivity and phase separation has been demonstrated in this work, the mechanisms by which it leads to toxicity need to be elaborated as detailed below.

Major points:
- Condensation can cause toxicity through various mechanisms, including concentration increase of the protein (1), concentration increase of nonspecific partners (2), mislocalisation (3), nonfunctional/toxic conformation stabilised by the condensate (4), promoting aggregation (4), sequestration (5), changes in posttranslational modifications (6). I suggest elaborating these points and correlations, where possible.
- In ref 7 the connection between dosage sensitivity and disorder is through promiscuous linear motifs. It would be useful to dissect the interactome part from phase separation, as condensate formation also leads to higher concentration of both specific and nonspecific partners.
- The experimental demonstration of how LoF mutations of dosage sensitive genes impact phase separation is a bit confusing. Overexpression can cause aggregation through solubility effects and formation of larger puncta is consistent with this.
- The section on rescuing phase separation compensating the LoF mutation effect is also misleading. The fusion of a 214 residue long disordered region can have a multitude of side-effects, in addition to phase separation.
- Based on several criticism on the field, a more stringent experimental analysis would be required to assess if phase separation is altered and aberrant (see papers by Robert Tjian and Andrea Mussacchio)
- I suggest to remove the arguments on the distinction between phase separating and non-phase separating TFs. Based on the earlier arguments by other authors that most TFs are disordered and disorder is required for phase separation, one expects that most TFs do phase separate.

Minor points
- For local effects, i.e. phase separating regions the authors should use only predictors trained for local effects (PScore for example was not trained to identify dropletpromoting regions). They should also note that most predictors were not trained to capture mutant effects.
- I suggest separating more clearly the arguments based on statistical analysis, prediction and novel experimental data.
- Elaborate the novelty of the present study with respect to ref 11

**Authors’ response**

Reviewer #1:

In this work Liang Yanga and collaborators investigated the relationship between gene dosage sensitivity and phase separation. The proposed hypothesis relies on the observation that upon reaching a certain critical concentration IDP tend to phase separate, forming biomolecular condensates that functionally compartmentalize biological function. However, the author did not consider two relevant parameters: the Csat varies from protein to protein, thus indicating that there is no clear evidence for which the decrease of the 50% of the protein level (for the haploinsufficiency) is sufficient to cross the critical threshold, thus affecting LLPS within cells. The other aspect relates to the fact that while the haploinsufficiency can globally reduce the protein abundance, the local protein concentration within the condensates may remain stable, arguing against a sensitivity to gene dosage. These aspects are not investigated nor discussed, thus limiting the relevance of the findings. We encourage the authors to properly address the contribution of the cellular microenvironments to the dynamic formation of condensates, related to the analyzed proteins. Although the proposed relationship is of relevance and the combined analytical strategy indeed suggest a possible correlation between sensitivity to gene dosage and the propensity to phase separate of the gene products, the conclusions are not always fully supported by the presented data. Indeed, there are important technical limitations that reduce the impact and the robustness of this study that needed to be addressed before considering the manuscript ready for publications.

Response: Thanks to the reviewer. These constructive criticisms have brought us profound thinking and corresponding improvements.

Major criticisms:

1. The droplet assays showed some relevant technical limitations, as they have been performed starting from desaturated recombinant proteins, whose re-folding/structure have not been measured. The obtained results could be strongly affected by the multiple protein conformations that the recProtein could have explored upon resuspension in the Droplet assay buffer. If this point can not be experimentally addressed, it would be necessary to repeat all the droplet assays using native recombinant proteins or properly re-folded one.

Response: We appreciate the reviewer's constructive criticism. The majority of examples in phase separation investigations focused upon intrinsically disordered proteins, as the case of SOX2, PAX6 and PQBP1 in our manuscript. The question of whether a certain conformation is adopted throughout the phase separation method has long been a source of debate. Evidences from Dr. Steven L. McKnight et al. showed that the intrinsically disordered regions of TDP43 self-assemble into a labile, cross-β polymeric conformation in phase separated liquid-like droplets (Proc Natl Acad Sci U S A. 2020 Nov 17;117(46):28727-28734.), and the similar cross-β polymeric conformation exist as in polymeric fiber as in solid state, in liquid-droplets in liquid state, and in the native hnRNPA2 protein presented in isolated nuclei (Cell. 2015 Nov 5;163(4):829-39.) However, another group of scientists stick with the idea that these specific proteins remain disordered all the way during phase separation. With that being said, indeed, our experiments started with denatured proteins without knowing their folding conformation, but this is a standard way of working with “intrinsically disordered proteins” such as FUS (Cell. 2017 Oct 19;171(3):615-627.e16.), Pbp1(Cell. 2019 Apr 18;177(3):697-710.e17.), and hnRNPA1 (Nat Commun. 2020 Dec 11;11(1):6349.). Due to the nature of these proteins, it is challenging to employ native recombinant or properly re-folded ones, and the issues of protein conformational changes during phase separation is beyond the scope of this study. We appreciate the reviewers for understanding.

2. For the droplet assay, once they will have address the previous comment, the authors should be careful in arguing of complex phase transition (glass- or solid-like states) without providing direct measurements of these specific states. In addition, they should motivate the reason why they are using such a broad range of protein concentrations. All the data lack any quantification and replicates, which strongly limits the relevance of these observations. Finally, the author should have included controls: they need to provide evidence that the observed behavior depends on the predicted IDR of each of the analyzed protein.

Response: Thanks to the reviewer for his/her suggestions. We have made the following improvements in response to the above suggestions:

a)We have carefully reconsidered the description of condensate status and made corresponding changes in the revised manuscript. We have removed the description of gel-like or solid-like state. See page 7 of the revised manuscript for details.

b)Concerning the reason why we used such a broad range of protein concentrations in droplet assay experiment, this approach is a common research method in the field of phase separation when exploring the phase separation ability of proteins (Nature. 2012 Mar 7;483(7389):336-40.; Cell. 2016 Jun 16;165(7):1686-1697.), so as to comprehensively explore phase separation phenomena in different states.

c)We are very sorry that we did not provide a clear description of quantification and replicates, which we have emphasized in the revised manuscript. Accurate quantification and replicates are indeed important to our work. Our experiments all provided appropriate quantification and replicates:

In the FRAP experiment of Figure 2C, we selected 3 droplets to repeat the measurement.

In the droplet observation experiments of Figure 3B-C, 4D, 4H, 4F, although we only showed the image of a single cell in the main figures, we showed the images of multi- view and multi-cell in the supplementary figures. In addition, we selected multiple cells (at least 6 cells) in multiple views (at least 3 views) and quantified the droplet properties of the cells, including puncta fluorescence intensity, puncta number, and puncta sum area. We displayed the counted cell numbers in the figures and made appropriate statistics test.

d)For the problem of the control groups, we added control groups including protein-IDR (intrinsically disorder region) and protein-△IDR droplet assays. These experiments proved that the phase separation phenomenon of HNRNPK, PQBP1 and PAX6 depends on the key region of phase separation (Fig. S6). See page 8 of the revised manuscript for details.

3. Related to Figure 3, the author performed genome editing with CRISPR/Cas9, but details are missing both tin the results and methods section. The talk about "knockdown" of SOX2 and PAX6 but this terminology does not mean much related to genome editing: either they generated KO cell lines or haploinsufficiency by targeting one of the two allele. They should provide infos on the kind of genetic perturbation they retrieved, the kind of mutations, using at least two independent cell line/gene to then perform the downstream analyses. For what concerns the IF measurements, it is not specified the numerosity of the analyses samples nor the number of biological replicates by which the used statistical test are not meaningful. Furthermore, if they are comparing WT vs haplo-insufficient cells, how do they justify that they measured a non- linear decrease of the IF intensity? The are no information about the normalization approach used for quantifying the IF intensity, please provide it.

Response: Thanks to the reviewer for the suggestion. We are very sorry that our description of the relevant details is not clear enough, we supplemented the relevant detailed descriptions in the revised results and methods:

a) We performed CRISPR/Cas9 heterozygous knockdown of each gene by targeting one of the two alleles.

b) We provided the sequencing results after gene editing in the revised supplementary figures (Fig. S7D-E). In the figures, we described the specific situation of heterozygous mutations. We used cells as test tube to provide the cellular environment for target proteins, which enables us explain the dosage-effect of the phase separation of HI proteins. Here we did not consider the influence of multiple biological backgrounds in different cell lines on the phase transition ability. Moreover, the HI genes we studied are sensitive to protein dosage. As the endogenous dosage of proteins in different cell lines varies greatly, using multiple cell lines with different levels of endogenous expression will create confusion, and introduce different biological context to this question. Therefore, we kept describing our findings with one cell line. One cell line with one biological background is suitable and sufficient to support our conclusion that PQBP1 and SOX2 phase separate in cells (Fig. 4D, H), and to help us discover the none-liner relationship between PQBP1 protein expression and phase separation properties (Fig. 3F-G). In future studies, such as the effect of different internal microenvironment on phase separation properties, we will use multiple cell lines to study the same gene. Hope this explains your confusion.

c) In the quantitative analysis of IF intensity of cells, we selected multiple cells (at least 150 cells) in multiple views (at least 3 views) for analysis (Fig. S8G-H). We described the number of statistical samples in figures (Fig. 3B-E).

d) For the normalization approach in quantifying the IF intensity, we described the details of fluorescence intensity detection and normalization with CellProfiler 3 and updated these descriptions in the revised method (Page 20).

For the question concerning how we justify that we measured a non-linear decrease of the IF intensity comparing wild-type with heterozygous knockdown cells, we normalized the phase separation intensity by the mean fluorescence intensity indicating protein concentration in the cells. The results showed that the normalized phase separation intensity in the knockdown cell lines is still lower than that of wild- type cells (Fig. 3D-E). This result indicated that in the heterozygous knockdown cells phase separation intensity decreases more dramatically than protein concentration, that is, the non-linear relationship. See page 9 of the revised manuscript for details.

4. The analyses of the relationship between mCherry-PQBP1 levels and the formation of droplet is of interest. However, the experiment misses relevant controls: what is the level of the mCherry-PQBP1, with respect to the endogenous level of PQBP1? The author should provide the same analyses using a PQBP1 lacking its IDR or relevant AA involved in LLPS (that can be determined using the in vitro droplet assay). Please address these points.

Response: Thanks to the reviewer for the suggestion. We agree with the reviewer that the absolute quantification of PQBP1 level is important, especially the concentration of mCherry-PQBP1 in relation to the endogenous level. Thus, we have revised the manuscript so as to better illustrate our point of view (Page 9). In point 3, we found that the relationship between changes in protein phase separation property and protein concentration gradient is non-linear when comparing wild-type with heterozygous knockdown cells (Fig. 3D-E). In order to verify whether the non-linear relationship still exists under continuous protein expression concentrations, we used the PQBP1 knock- out cells as test tube and complement mCherry-PQBP1 to draw the relationship between phase separation property and protein concentration. Since fixation may lead to artifacts by changing the appearance of puncta sizes and numbers (J Phys Chem B. 2023 May 18;127(19):4165-4173, Elife. 2022 Nov 29;11:e79903), we used live-cell imaging and detected mean fluorescence intensity to examine the protein concentration of mCherry-PQBP1. The results showed that the intensity of mCherry- PQBP1 puncta fluorescence displays a non-linear relationship with the concentration of transfected protein (Fig. 3F). Despite our attempts, we failed to generate mCherry knock-in cell line, which limits our in-depth comparison of endogenous PQBP1 level and each individual mCherry-PQBP1 level. We are also willing to find more precise quantitative methods in future investigations to make our conclusions more robust. For the control question, we added PQBP1-△IDR as a negative control and we found no correlation between the formation of puncta and mCherry-PQBP1-△IDR protein level (Fig. 3G). See page 9 of the revised manuscript for details.

5. Related to the results presented in Figure 4D: the protein level of the WT or p.Gly129fs mCherry-SOX2 should be included to address whether the observed differences are related to the protein abundance. Once again, please compare these protein levels with respect to the endogenous SOX2 and provide quantitative values (with replicates) to strengthen the robustness of these data. Same issue for results presented in Figure 4G/H.

Response: Thanks to the reviewer for the suggestion. We made the following improvements in response to the above suggestions:

a)In order to measure the protein concentration in each cell, we detected the mean fluorescence intensity in cells to represent the protein concentration and compared the protein levels of the WT or p.Gly129fs mCherry-SOX2 in Figure 4D or PQBP1 in Figure 4H. We found that the difference in the mean fluorescence intensity among cell lines was subtle, i.e. the protein concentration has little variations (Fig. S10B, D). For your concerns about replicates, we have provided the results of repeated experiments including multi-view (at least 3 views) and multi-cell (at least 6 cells) in Figure S10 in the original manuscript. We described the number of statistical samples in figures. The droplet properties were sufficiently quantified, including puncta fluorescence intensity, puncta number, and puncta sum area.

b)For the issue of comparing these protein levels with respect to the endogenous SOX2, we agree that having the endogenous SOX2 levels as control is of great significance in a number of experiments. Nevertheless, our purpose of overexpressing mutant proteins is to observe the effect of the mutation on phase separation properties. For instance, Steven L. McKnight et al. (Science. 2022 Jul;377(6601):eabn5582.) overexpressed GFP-tagged TDP-43 variants in cultured cells to observe the puncta and test phase separation properties. This suggests that such qualitative experiment has been used in the field of phase separation before. In addition, we have also considered the relationship between endogenous protein dosage and phase separation. We performed immunofluorescence staining to compare SOX2 protein levels and phase separation properties in wild-type and heterozygous knockdown cells (Fig. 3B-E). The results showed that the fluorescence intensity of SOX2 puncta decreased when the endogenous protein level decreased.

6. Referring to Figure 5, the authors attempted to evaluate the effects of altered LLPS on cellular phenotype, namely changes in the gene expression pattern of downstream targets of TFs harboring IDRs (predicted to go through LLPS). The computational prediction of the TF-regulated genes is not appropriate as the used criteria have not been verified/tested by any means. Indeed, selecting the top 50 differentially expressed genes by using the mean expression of cancer datasets retrieved from patients not carrying the PTVs for the analyzed TFs is rather an erroneous assumption, as it would imply that the difference in the gene expression pattern of these group of genes will solely depends on the alteration of the in silico predicted TFs. It is well known that cancers result by the combination of multiple genetic, epigenetic and environmental alterations and assuming the differences are depending on a single mutation of a TFs is simply incorrect. In addition, the author did not provide any supportive data indicating the robustness of the in silico methodology that has been adapted. Therefore, all the obtained analyses do not support the raised conclusions.

Response: In order to facilitate the bioinformatics analysis, we simplified the model of transcription factor without further consideration of other complex factors. We admitted that our model was too simplified, and the method of measuring the intensity of downstream target gene expression has not been fully verified. We deleted this part from the article and added analysis based on perturb-seq single-cell data (Fig. 5A-B). In the original manuscript, we limited the analysis of perturb-seq data to TFs. In the revised manuscript, we extended the analysis to all genes in the dataset. The results demonstrated that LoF genetic perturbations on phase-separating genes cause similar transcriptional phenotypes as those on dosage-sensitive genes, suggesting that perturbation of phase-separating genes results in dosage-sensitive-like effect. See pages 11-12 of the revised manuscript for details.

7. Referring to the attempt of experimentally demonstrate that the IDRs of TFs is necessary for their biological function, the author analyzed the puncta distribution and the gene expression activity of a reconstituted SOX2-FUS chimeric protein (Figure 5 F- G). However, the obtained data argue against their conclusion as the luciferase assay showed clearly that the chimeric TF is 10 time less efficient in inducing gene expression. Although this result is not per se surprising, it does not argue in favors of the author conclusion, which should be further supported by analyzing the effects of rescuing the SOX2 KD with the chimeric protein (with respect to the WT one) on the expression of endogenous SOX2-targets. In addition, is the chromatin binding and the residence time of its endogenous target maintained? Many controls are missing to reach such a conclusion.

Response: Thanks to the reviewer for the suggestion. We performed RT-qPCR experiment for downstream functional analysis after rescuing the SOX2 heterozygous knockdown cell line with the SOX2-FUS chimeric protein on the expression of endogenous target genes. We performed two batches of experiments, each containing 3 replicates. We found that as with the wild-type SOX2, the FUS-IDR fused SOX2- p.Gly129fs improved expression of endogenous target genes in heterozygous knockdown cells for most of all SOX2 activated target genes in TRRUST database (Fig. 5E, Fig. S11). See pages 12-13 of the revised manuscript for details.

Reviewer #2:

The manuscript by Dr Li and colleagues addresses an important problem and aims to decipher the molecular basis of dosage sensitivity using phase separation as a possible mechanism. This problem has been addressed before in PMID 27320918 (ref 11), where the link between concentration dependent cellular toxicity and liquid-liquid phase separation (LLPS) has been suggested.

Although the connection between dosage sensitivity and phase separation has been demonstrated in this work, the mechanisms by which it leads to toxicity need to be elaborated as detailed below.

Response: We thank the reviewer for his/her specific comments.

Major points:

1- Condensation can cause toxicity through various mechanisms, including concentration increase of the protein (1), concentration increase of nonspecific partners (2), mislocalisation (3), nonfunctional/toxic conformation stabilised by the condensate (4), promoting aggregation (4), sequestration (5), changes in posttranslational modifications (6). I suggest elaborating these points and correlations, where possible.

Response: Thanks to the reviewer for the suggestion. In response to this issue, we have added corresponding content in the discussion section (Page 15). PMID 27320918 focuses on the dosage sensitivity of over-expression of phase-separating proteins, suggesting that excessive aggregation of phase-separating proteins can be toxic to cells. Our paper mainly focuses on the dosage sensitivity caused by the under- expression of phase-separating proteins. We are also very interested in exploring how the overexpression of phase-separating proteins causes cytotoxicity. However, considering the lack of existing data on phase-separating protein overexpression and the limitations of existing experimental techniques, this issue is difficult to investigate in depth at this stage.

2- In ref 7 the connection between dosage sensitivity and disorder is through promiscuous linear motifs. It would be useful to dissect the interactome part from phase separation, as condensate formation also leads to higher concentration of both specific and nonspecific partners.

Response: We agree with the reviewer that we need to dissect the interactome part in the disordered regions from phase separation. In order to exclude the possibility that promiscuous linear motifs in the disordered regions might be the cause of the association between phase separation and dosage sensitivity, we compared the dosage sensitivity score between the known phase-separating proteins with high and low content of disordered regions. Compared to proteins in the human proteome, the scores for dosage sensitivity were significantly higher both for phase-separating proteins with high and low content of disordered regions (Fig. 1G). Therefore, this result demonstrated that dosage sensitivity is highly correlated with phase separation independent of disordered regions. See pages 6-7 of the revised manuscript for details.

3- The experimental demonstration of how LoF mutations of dosage sensitive genes impact phase separation is a bit confusing. Overexpression can cause aggregation through solubility effects and formation of larger puncta is consistent with this.

Response: Thanks to the reviewer for the suggestion. We agree that detecting proteins at endogenous levels are important, due to the fact that phase separation is a highly dosage-dependent procedure.

a) Overexpression is a well-accepted tool to study phase separation in cells. Peter St George-Hyslop et al. (Cell. 2018 Apr 19;173(3):720-734.e15.) investigated FUS phase separation in SH-SY5Y cells by transiently expressing YFP-tagged FUS. They studied the changes in the number of cells displaying intracellular FUS variants granules. In addition, Douglas H. Phanstiel and Gang Greg Wang et al. (Nature. 2021 Jul;595(7868):591-595.) stably overexpressed GFP-tagged NUP98 truncated proteins and chimeras, which helped them define the role IDR and its potential phase separation plays in tumorigenicity. Steven L. McKnight et al. (Science. 2022 Jul;377(6601):eabn5582.) also transfected cultured cells with GFP-tagged TDP-43 variants to study their puncta. J Paul Taylor et al. (J Cell Biol. 2021 Mar 1;220(3):e202009079.) transfected U2OS cells with GFP-NPM1 to detect the mobility of NPM1 granules.

b) It is true that overexpression may bring about unorthodox results, and we have also used other ways to prove that target protein does indeed phase separate. In order to investigate the phase separation ability of SOX2 and PQBP1 LoF mutants, we also purified the corresponding proteins and performed droplet formation assay in vitro (Fig. 4C-D, G-H).

In addition, we acknowledge that construction of knock-in cell lines is a better alternative than overexpression. In our experiments, we also made attempts to generate fluorescence-labeled knock-in cell line, but unfortunately it didn't work out. Therefore, we adopted alternative strategy of using knockout or knockdown cells and rescued them with wild-type or mutant proteins. We are cautious over the amount of rescuing proteins added to avoid overexpression, which might disrupt the original cellular functions. We provided the results of these experiments including multi-view and multi-cell in Figure S10, and the puncta properties were quantified, including puncta fluorescence intensity, puncta number, and puncta sum area, which makes our experimental results more reliable.

4- The section on rescuing phase separation compensating the LoF mutation effect is also misleading. The fusion of a 214 residue long disordered region can have a multitude of side-effects, in addition to phase separation.

Response: Thanks for pointing out the issue. Your concerns are valid, and we have taken them into account. FUS-IDR has been thoroughly studied and many researchers have chosen it to rescue the phase separation. It’s common to replace protein phase- separating domain with an orthogonal one to demonstrate their functions, and FUS- IDR is widely used because it has very few side-effects. Wenyu Wen et al. (Nat Commun. 2020 May 8;11(1):2266.) used FUSL-Par3N chimera to study the mechanism of NTD-mediated Par clustering. Kang Shen et al. (Nature. 2020 Dec;588(7838):454-458.) replaced SYD-2’s IDR with well-studied phase separation motifs of human FUS to rescue the SYD-2(IDR∆) mutant. Moreover, Patrick Cramer and Ritwick Sawarkar et al. (Mol Cell. 2021 Mar 4;81(5):1013-1026.e11.) found that the IDR in NELFA, which was necessary for nuclear NELF condensation, could be functionally replaced by the IDR of FUS protein. To further demonstrate that phase separation of SE requires an N-terminal IDR, Pilong Li and Yijun Qi et al. (Nat Cell Biol. 2021 Jan;23(1):32-39.) fused the LCD of FUS to the N terminus of SE∆IDR1. In all of these studies, FUS-IDR was able to successfully rescue the phase separation and no other anomalies were produced.

In addition, we performed RT-qPCR experiment for downstream functional analysis after rescuing the SOX2 heterozygous knockdown cell line with the SOX2-FUS chimeric protein on the expression of endogenous target genes. We found that as with the wild-type SOX2, the FUS-IDR fused SOX2-p.Gly129fs successfully improved expression of endogenous target genes in heterozygous knockdown cells for most of all SOX2 activated target genes in TRRUST database (Fig. 5E, Fig. S11). See pages 12-13 of the revised manuscript for details.

5- Based on several criticism on the field, a more stringent experimental analysis would be required to assess if phase separation is altered and aberrant (see papers by Robert Tjian and Andrea Mussacchio)

Response: Thanks to the reviewer for the suggestion. We agree with the reviewer that we need stringent experimental analysis. Due to technical limitations, there are indeed some imperfections in the current field of phase separation. We noticed Robert Tjian et al. (Genes Dev. 2019 Dec 1;33(23-24):1619-1634.) emphasized that the in vivo experiment of phase separation required rigorous quantitative analysis and consideration. Andrea Mussacchio et al. (EMBO J. 2022 Mar 1;41(5):e109952.) clarified the complexity of phase separation and incomplete tests of many phase separation claims. To solve the above problems, we carried out multi-party verification as much as possible, including in vitro phase separation experiment, immunofluorescence staining, cell overexpression experiment, etc., and arranged suitable controls. We also used software for identifying cell puncta as well as appropriate statistical tests to carry out fine quantification of our cell imaging, in order to minimize the artifacts and avoid mistakes in our experiment.

6- I suggest to remove the arguments on the distinction between phase separating and non-phase separating TFs. Based on the earlier arguments by other authors that most TFs are disordered and disorder is required for phase separation, one expects that most TFs do phase separate.

Response: Thanks for pointing out the issue. We acknowledged that most TFs have the potential to phase separate, so we no longer distinguish between phase separating and non-phase separating TFs in the revised manuscript. Therefore, we deleted the TF model based on TCGA-omics data and extend perturb-seq data analysis (Fig. 5A-B). In the original manuscript, we limited the analysis of perturb-seq data to TFs. In the revised manuscript, we extended the analysis to all genes in the dataset. The results demonstrated that LoF genetic perturbations on phase-separating genes cause similar transcriptional phenotypes as those on dosage-sensitive genes, suggesting that perturbation of phase-separating genes results in mimics dosage-sensitive effect. See pages 11-12 of the revised manuscript for details.

Minor points

- For local effects, i.e. phase separating regions the authors should use only predictors

trained for local effects (PScore for example was not trained to identify droplet promoting regions). They should also note that most predictors were not trained to capture mutant effects.

Response:

We reconsidered the features used in the TruncPS model and deleted features that were not belonging to the phase separation local effects, such as PScore and PLAAC features. After modification, the prediction performance of new version of TruncPS was still much better than currently available phase separation predictors used to screen phase-separation-prone regions (Fig. 4A, Fig. S9).

- I suggest separating more clearly the arguments based on statistical analysis, prediction and novel experimental data.

Response: We are very sorry for our unclear elaboration. We have improved the compatibility of different parts of the content and improved the description.

- Elaborate the novelty of the present study with respect to ref 11

Response: Thanks for the suggestion. We complemented the novelty between our paper and PMID 27320918 in discussion part. It mainly includes the following aspects:

a) PMID 27320918 focuses on the dosage sensitivity of over-expression of phase- separating proteins, suggesting that excessive aggregation of phase-separating proteins can be toxic to cells. However, our paper mainly focuses on the dosage sensitivity caused by the under-expression of phase-separating proteins. b) The dosage-sensitive gene set used in PMID 27320918 is derived from yeast. This paper focuses on the dosage-sensitive gene set that causes disease in humans. c) This paper more systematically expounds the relationship between dosage sensitivity and phase separation, and deeply explores the impact of abnormal phase separation on downstream functions. See page 15 of the revised manuscript for details

**2^nd^ round**

**Reviewer 1**

I acknowledge that the Authors did numerous additive experiments and analyses in the attempt to address the raised criticisms.
However there are still some critical points that for which they did not provide novel data/explanation that strongly limits the relevance of the drawn conclusions.

a. In response to our criticisms in point 3. Related to the necessity to provide an independent clone carrying the haploinsufficiency of the investigated genes, the authors stated that "One cell line with one biological background is suitable and sufficient to support our conclusion".
I strongly disagree with this assumption as the CRISPR/Cas9 methodology, which is based on clonal selection and genome editing, can cause uncontrolled biological and genetic (off-targets) effects for which it is mandatory to repeat some of the key experiment using an independent clone.
b. Regarding the comments on point 4 and 5, in which we asked to compare the level of the mCherry-fused protein with the endogenous level, these data were not provided. This again is still a critical aspect as they are looking to dosage sensitivity for which a controlled protein level of the experimental system is necessary. The justification that in other papers this kind of qualitative assays have been used does not mean anything, considering the biological question that the authors intended to address. They must provide quantitative valued of the chimeric protein levels, compared to the endogenous one. This can be easily defined by different means, including quantification of immunoblot (in biological replicates) or MS.
We understand that obtaining the same data on the endogenous protein is not trivial (by Knock-in approach), but we did not ask for such a kind of experiments. We simply ask for a comparative analyses with respect to the endogenous protein, and also the Authors agreed on the relevance of this analyses ("For the issue of comparing these protein levels with respect to the endogenous SOX2, we agree that having the endogenous SOX2 levels as control is of great significance in a number of experiments").

These experiments are mandatory to properly support the raised conclusions.

**Authors’ response**

Reviewer #1: I acknowledge that the Authors did numerous additive experiments and analyses in the attempt to address the raised criticisms.

However there are still some critical points that for which they did not provide novel data/explanation that strongly limits the relevance of the drawn conclusions.

Response: Thanks to the reviewer. These constructive criticisms have brought us profound thinking and corresponding improvements.

a. In response to our criticisms in point 3. Related to the necessity to provide an independent clone carrying the haploinsufficiency of the investigated genes, the authors stated that "One cell line with one biological background is suitable and sufficient to support our conclusion".

I strongly disagree with this assumption as the CRISPR/Cas9 methodology, which is based on clonal selection and genome editing, can cause uncontrolled biological and genetic (off-targets) effects for which it is mandatory to repeat some of the key experiment using an independent clone.

Response: We greatly appreciate the constructive criticism provided by the reviewer. There was a misunderstanding on our part regarding the reviewer’s request for multiple clones from two independent cell lines. We attempted genetic manipulation in various cell lines, including U2OS and HEK 293T, among others. However, only the HEK 293T cell line exhibited a successful CRISPR heterozygous knockdown effect. Therefore, in our initial manuscript, we only presented data from one clone cell line for SOX2 and PAX6.

We acknowledge the reviewer's concern regarding the potential high off-target rate associated with CRISPR/Cas9 methodology. Therefore, in the revised manuscript, we have included the experimental results and statistical analysis from two independent heterozygous knockdown clonal cell lines for each gene (Fig. 3B-E, Fig. S8G-J). These results demonstrate that the observed experimental phenomena and statistical outcomes remain consistent across both clonal cell lines. Moreover, we have provided the mutational information of the two independent clonal cell lines in Fig. S8D-E.

b. Regarding the comments on point 4 and 5, in which we asked to compare the level of the mCherry-fused protein with the endogenous level, these data were not provided. This again is still a critical aspect as they are looking to dosage sensitivity for which a controlled protein level of the experimental system is necessary. The justification that in other papers this kind of qualitative assays have been used does not mean anything, considering the biological question that the authors intended to address. They must provide quantitative valued of the chimeric protein levels, compared to the endogenous one. This can be easily defined by different means, including quantification of immunoblot (in biological replicates) or MS.

We understand that obtaining the same data on the endogenous protein is not trivial (by Knock-in approach), but we did not ask for such a kind of experiments. We simply ask for a comparative analyses with respect to the endogenous protein, and also the Authors agreed on the relevance of this analyses ("For the issue of comparing these protein levels with respect to the endogenous SOX2, we agree that having the endogenous SOX2 levels as control is of great significance in a number of experiments").

Response: Thanks to the reviewer for the suggestion. We concur with the reviewer's viewpoint, emphasizing the significance of considering the concentration of mCherry- fused proteins relative to the endogenous levels. In response to this feedback, we have incorporated the western blot results of mCherry-fused proteins alongside the endogenous proteins in the revised manuscript (Figure S11). These results demonstrate that the expression levels of diverse mCherry-fused proteins of PQBP1 are essentially comparable to the endogenous PQBP1 levels (Figure S11 A-B). However, the expression levels of various types of mCherry-fused proteins of SOX2 were observed to be higher than the expression levels of endogenous SOX2 (Figure S11 C). Actually, we investigated the expression levels of various mCherry-fused SOX2 proteins in HEK 293T cells by transfecting different amounts of plasmids and subsequently performing western blot analysis to compare the overexpressed proteins with the endogenous SOX2 proteins. The obtained results provided insights into the relative expression levels under different transfection conditions. Specifically, we found that the expression levels of mCherry-fused SOX2 proteins derived from 1 ng plasmids were comparable to that of the endogenous SOX2 (Figure S11 D). But because of the inherently low expression level of the SOX2 protein in the HEK 293T cell line, supplementing mCherry-fused SOX2 to match the levels of endogenous SOX2 proved insufficient for imaging purposes.

Considering the specific requirements for imaging and taking into account transfection efficiency, we provided the results of transfection with two distinct plasmid amounts, namely 5 ng and 50 ng, for subsequent imaging experiments. Notably, transfection with 5 ng plasmids yielded a fluorescence signal that was relatively weak, hindering the formation of a clear image. Conversely, transfection with 50 ng plasmids resulted in pronounced fluorescence signals, enabling the visualization of clear images and punctate structures (Figure S11 E). Considering the experimental objective of comparing the phase separation properties of different SOX2 fusion proteins, we decided to transfect 50 ng plasmids of mCherry-fused SOX2. While this level of transfection led to expression levels surpassing the endogenous SOX2 protein, it guaranteed the quality of live cell imaging experiments, thereby facilitating the examination of the desired phase separation phenomena.

These experiments are mandatory to properly support the raised conclusions.

Response: We thank the reviewer for his/her specific comments.

**3^rd^ round**

**Reviewer 1**

The Authors addressed the raised critisms, providing more data about the expression level, which are still not fully satisfying the issue for SOX2, but at least they provided the requested comparison.
